# Origin of unusual HREE-Mo-rich carbonatites in the Qinling orogen, China

**DOI:** 10.1038/srep37377

**Published:** 2016-11-18

**Authors:** Wenlei Song, Cheng Xu, Martin P. Smith, Jindrich Kynicky, Kangjun Huang, Chunwan Wei, Li Zhou, Qihai Shu

**Affiliations:** 1 Laboratory of Orogenic Belts and Crustal Evolution, School of Earth and Space Sciences, Peking University, Beijing 100871, China; 2 School of Environment and Technology, University of Brighton, Brighton BN41 2HQ, United Kingdom; 3 Department of Geology and Pedology, Mendel University, Brno 61300, Czech Republic; 4 Key Laboratory of High-temperature and High-pressure Study of the Earth’s Interior, Institute of Geochemistry, Chinese Academy of Sciences, Guiyang 550002, China; 5 State Key Laboratory of Geological Processes and Mineral Resources, China University of Geosciences, Beijing 100083, China

## Abstract

Carbonatites, usually occurring within intra-continental rift-related settings, have strong light rare earth element (LREE) enrichment; they rarely contain economic heavy REE (HREE). Here, we report the identification of Late Triassic HREE-Mo-rich carbonatites in the northernmost Qinling orogen. The rocks contain abundant primary HREE minerals and molybdenite. Calcite-hosted fluid inclusions, inferred to represent a magmatic-derived aqueous fluid phase, contain significant concentrations of Mo (~17 ppm), reinforcing the inference that these carbonatitic magmas had high Mo concentrations. By contrast, Late Triassic carbonatites in southernmost Qinling have economic LREE concentrations, but are depleted in HREE and Mo. Both of these carbonatite types have low δ^26^Mg values (−1.89 to −1.07‰), similar to sedimentary carbonates, suggesting a recycled sediment contribution for REE enrichment in their mantle sources. We propose that the carbonatites in the Qinling orogen were formed, at least in part, by the melting of a subducted carbonate-bearing slab, and that 10 Ma younger carbonatite magmas in the northernmost Qinling metasomatized the thickened eclogitic lower crust to produce high levels of HREE and Mo.

China currently supplies 85% of the World’s rare earth elements (REE)^1^,^2^, however this supply is expected to decrease as China reduces REE exports. Economically heavy REE (HREE: Gd-Lu + Y)-rich mineralization is overall less common than light REE (LREE: La-Eu)-rich examples. Due to the range of applications of HREE, for example in green technologies, demand for these elements is high^2^. Nearly all HREE are sourced from granite-related HREE-enriched residual clays in South China^1^. Worldwide exploration is now leading to the deployment of a rare earth supply chain outside China. For instance, the EURARE project has been funded to explore for REE resources in Europe, in which the most important resources are associated with alkaline igneous rocks and carbonatites, such as Sarfartoq and Qaqarssuk (Greenland), Fen (Norway)^2^. Carbonatites are mostly situated in anorogenic settings related to intra-continental rifting^3^, and they frequently occur in ring-complexes associated with alkaline silicate rocks^4^,^5^. Carbonatites are important hosts for LREE resources worldwide^3^. They rarely contain HREE minerals, with the main example derived from alteration of early-formed HREE-rich zircon^6^. The carbonatites in the northernmost Qinling (NQ) orogenic belt (Fig. 1) contain quartz and economic molybdenite which are quite rare, and they show relatively flat REE patterns with high HREE contents (e.g., Yb > 30 ppm)^7^,^8^. These mineralogical and geochemical characteristics distinguish them from almost all reported carbonatites.

Three major petrogenetic models have been previously proposed for the formation of carbonatites: (1) melting of carbon-bearing mantle source rocks^9^; (2) immiscibility from CO_2_-saturated silicate melts^10^; and (3) residue formed via fractional crystallization of carbonated silicate melts^11^. Experimental studies have shown that primary carbonatitic melts produced by a low degree of partial melting are LREE-enriched and depleted in HREE and Si^12^,^13^, consistent with carbonatitic melt inclusion studies^14^. Experimental and natural melt inclusion studies have demonstrated that HREE and Si will preferentially partition into conjugate silicate melts relative to the carbonate melt during immiscibility (D^La/Lu^_carbonate/silicate melts_ = 1.3–4.5)^15^,^16^. Crystal fractionation of dense silicates^17^ and oxides (such as REE-enriched perovskite and pyrochlore) from carbonatites could decrease the HREE and Si contents of the residual magmas, depending on the mineral composition. Therefore, none of the three petrogenetic models noted above is likely to be responsible for significant HREE enrichment in carbonatites.

Here we present observations on primary HREE minerals and Mo-containing fluid inclusions in the NQ carbonatites, and Mg isotope systematic, obtained in order to constrain carbonatite origin. The Qinling orogenic belt separates the North China Block from the South China Block (Fig. 1a). Closure of the Mianlue Ocean in the Triassic resulted in collision of the South China Block with the Qinling belt along the Mianlue suture, followed by amalgamation of the South and North China Blocks^18^. The NQ is a world-famous province for Mo ore production: six giant Mo deposits and numerous large, medium and small Mo deposits are present in this region^19^. The majority of the Mo deposits formed during the Late Jurassic to Early Cretaceous (148-112 Ma), and are closely associated with granitic porphyries^19^. The NQ carbonatite samples studied in this investigation were collected from Huayangchuan and Huanglongpu (Fig. 1b). The carbonatites occur as dykes and lenses, extending from tens of meters to over one kilometer, intruding into Late Archean gneiss and Mesoproterozoic andesitic rocks^8^. The carbonatites contain abundant molybdenite and form a large Mo deposit^7^.

## Results

### Primary REE minerals

The carbonatites examined in this investigation are, according to field observation and thin section evaluation, composed predominantly of calcite (90–95 volume %) and quartz (up to 5 volume %). Minor and accessory phases include barite, celestine, K-feldspar, augite, arfvedsonite, phlogopite, biotite, albite, sulfides, REE minerals, titanite and brannerite-(Fe). Molybdenite and REE minerals are characteristic minerals in this rock. Molybdenite occurs as irregular, thin, platy crystals or aggregates with galena, barite and REE minerals, embedded into aggregates of calcite (Fig. 2a–d and Supplementary Fig. S1a,c,d). The LREE minerals, consisting of bastnäsite-(Ce), parisite-(Ce), monazite-(Ce), allanite-(Ce), synchysite-(Ce) and fluorapatite, occur as aggregates with molybdenite + galena or aegirine-augite + phlogopite + ilmenite (Supplementary Fig. S1c,d,e). Synchysite-(Ce) co-exists with barite forming hexagonal-looking pseudomorphs, possibly of burbankite (Supplementary Fig. S1f), a mineral which was also observed as a trapped mineral in fluid inclusions in quartz (see below). Burbankite is generally considered to be an early REE mineral in carbonatites, and directly crystallized from an alkali-Sr-Ba-REE-rich carbonatitic melt^20^. Late hydrothermal fluids may however readily alter this minera^21^.

Primary HREE minerals were found in the carbonatites (Fig. 2) and have been grouped into xenotime-(Y) and Y-, Y(Ce)-Ca- and Y-Fe- silicate minerals in terms of their main element contents measured by EPMA (Supplementary Table S1). Xenotime-(Y) (Y_0.97–0.99_PO_4_) occurs as irregular to subhedral crystal intergrowths with molybdenite and monazite-(Ce) (Fig. 2a,b), or allanite-(Ce) (Fig. 2e). The Y-silicate mineral [(Y,Ce,Ca)_1.9–2.1_Si_2–2.1_O_7_·H_2_O] is potentially a hydrated form of keiviite-(Y), and forms small and parallel plate-like aggregates together with molybdenite and monazite-(Ce) (Fig. 2c,d). It is high in Y_2_O_3_ (~30 wt.%) and contains minor amounts of LREE (Ce_2_O_3_ = 3–6 wt.%). Minor CaO (0.2–3 wt.%) was also detected. This mineral shows a relatively constant REE distribution pattern on chondrite normalized (CN) plots and a low La/Yb_CN_ ratio of 0.39 (Fig. 3). The Y(Ce)-Ca-silicate minerals are potentially forms of britholite-(Y) and kainosite-(Ce) [(Y,Ce,Ca)_4.7–5.2_((Si,P)_1–1.1_O_4_)_3_F_0.7–1_ and Ca_0.8–1.1_(Ce,Y)_2.1–2.6_(Si_1.2–1.3_O_4_)_3_F_0.5–0.7_], and generally occurs as laminar crystals along the margins of the quartz. Small augite crystals were also observed in the quartz (Fig. 2f,g). The HREE-silicate mineral has variable contents of Y_2_O_3_ (5–22 wt.%) and CaO (4–14 wt.%) and elevated levels of LREE (Ce_2_O_3_ up to 18 wt.%). It contains minor amounts of F (up to 2.6 wt.%) and shows a variable REE pattern with La/Yb_CN_ ranging from 1.5–22. The Y-Fe-silicate mineral is potentially a hydrated form of rowlandite-(Y), and occurs as isolated platy crystals embedded in the calcite matrix (Fig. 2h). The core [(Y,Ce)_3.4–4.0_Fe_1.3–1.6_(Si_2.1_O_7_)_2_F_0.1–0.2_·H_2_O] has higher contents of FeO (~10 wt.%), LREE (Ce_2_O_3_ ~13 wt.%) and lower compositions of HREE (Y_2_O_3_ = 5–10 wt.%) than the rim (FeO ~5 wt.%; Ce_2_O_3_ ~4 wt.%; Y_2_O_3_ ~19 wt.%) [(Y,Ce)_3.9–4.5_Fe_0.8–1.0_(Si_1.9–2.2_O_7_)_2_F_0.02–0.03_·H_2_O]. Results from the rim show relatively stronger HREE enrichment (La/Yb_CN_ ~0.4). The hydration of these minerals is currently inferred from low analytical totals and requires further investigation.

### Mo and Si enrichment

The intergrowth of molybdenite with LREE and HREE minerals indicates contemporaneous formation. Monazite ages were determined in this study, yielding weighted mean U-Pb and Th-Pb ages of 208.9 ± 4.6 Ma and 213.6 ± 4.0 Ma, respectively (Supplementary Fig. S2 and Supplementary Table S2). These ages are close to the Re-Os age (ca. 221 Ma)^22^ of molybdenite in the carbonatites, and they are different to those of the granite porphyries and associated Mo deposits, which formed during the Late Jurassic to Early Cretaceous in the NQ orogen^19^. These results suggest that the Mo enrichment is a primary feature of the carbonatite magmas, as is also supported by the fluid inclusion results. Calcite, anhydrite, mirabilite, celestine and burbankite were determined as daughter minerals in the primary fluid inclusions in the calcite and quartz, this being consistent with the trapping of a carbonatite-exsolved fluid at subsolidus conditions (Supplementary Figs S3 and S4). Small but significant amounts of Mo (16 and 17 ppm; Supplementary Table S3) were determined in the calcite-hosted fluid inclusions by *in-situ* laser ablation ICPMS analysis. This concentration of Mo is comparable to that of fluid inclusions within the mineralized system from the super-large porphyry Mo deposit at Questa (6 to 90 ppm)^23^. To our knowledge this is the first case of detection of Mo in carbonatitic fluids.

Besides molybdenite and HREE minerals, the presence of abundant quartz in the NQ carbonatites distinguishes them from the majority of carbonatites worldwide. The origin of some quartz-bearing carbonatites has been postulated as representing calc-silicate skarn (pseudo-carbonatite)^24^,^25^. However, our chronology, mineralogy and geochemistry do not support such a pseudo-carbonatite origin for the rocks. There are no sediments and contemporaneous silicate rocks spatially associated with the carbonatites. Most of the granitic porphyries in the NQ formed during the Late Jurassic to Early Cretaceous (148–112 Ma)^19^. Burbankite, generally a diagnostic mineral of the carbonatites^20^, was identified in the studied rocks. Reaction between silicate magmas and Ca-carbonate would form wollastonite as experiments suggest^24^ and as is also seen in many skarn deposits^26^, while this mineral was not found in the rocks. Primary fluorapatites, observed in the carbonatites (Supplementary Fig. S1c), have high Sr and REE contents (Sr: >5100 ppm; total REE: up to 1.3 wt.%; Supplementary Fig. S5 and Supplementary Table S4). They show a strong LREE and HREE fractionation pattern (La/Yb_CN_ = 1.3–8.4) and a negligible Eu anomaly. These results are similar to those from other carbonatites worldwide^27^,^28^,^29^, but different to apatites in granitoids from various geographically occurrences^29^. Similarly, high Sr contents were determined in the carbonatitic calcites^30^, consistent with carbonates of a carbonatitic origin^27^,^28^.

The NQ carbonatites have C and O isotope compositions similar to normal carbonatites^7^, and they show Sr-Nd isotope ratios typical of enriched mantle sources^31^. Sulfur isotope data further support a mantle origin for the rocks (Supplementary Table S5). Molybdenite, galena and pyrite from the carbonatites display δ^34^S values varying from −6.7 to −7.7‰, −8.9 to −10.5‰ and −6.5 to −7.2‰, respectively. They are distinct from barite (4.6 to 5.1‰). As sulfur isotopes can be re-distributed between oxidized and reduced species during precipitation^32^, the actual δ^34^S (δ^34^S_ΣS_) of the carbonatitic liquids cannot be represented by the mean δ^34^S values of either sulfides or sulfates in this case. The δ^34^S_ΣS_ of the carbonatitic liquids has been calculated using galena and co-genetic barite as a mineral pair and using the δ-δ diagram^33^ (Fig. 4). As estimated from the intersection of a straight line fit to the data with the line of unit slope which shows no sulfide-sulfate fractionation (Δ = 0‰), the δ^34^S_ΣS_ value of the precipitating liquids is estimated to be around 1‰, similar to the mantle-derived value^34^.

### Mg isotopic compositions

Carbon isotopes are commonly used to trace subducting sediments^35^. Although they are effective in identifying recycled organic carbon, they are not sensitive to inorganic carbon which dominates subducted materials^36^. Recently, due to the fact that subducting lithologies underwent little Mg isotope fractionation during metamorphic dehydration and magmatic processes^37^,^38^, Mg isotope systematics have been applied to trace recycled sediments. The Mg isotopic compositions of carbonatites from the NQ, and Miaoya (233 Ma) in the southernmost Qinling orogen are reported in Table 1. The carbonatites in the southernmost Qinling host a large LREE deposit^39^. Both carbonatites have significantly lower Mg isotope ratios than typical mantle derived rocks, including Mid Ocean Ridge Basalt (MORB), Ocean Island Basalt (OIB) and peridotite xenoliths (−0.25 ± 0.07‰)^37^, as well as intra-continental rift-hosted carbonatites from Oldoinyo Lengai (0.13–0.37‰)^40^, with δ^26^Mg ranging from −1.28 to −1.07‰ and −1.89 to −1.37‰ at NQ and Miaoya, respectively (Fig. 5).

Magnesium isotopic fractionation can result from a range of processes, including mantle melting, crystal fractionation, silicate-carbonate liquid immiscibility and crustal contamination. There is insignificant Mg isotopic fractionation during partial melting of the mantle, or from olivine and pyroxene crystallization^37^,^40^,^41^. Li *et al*.^40^ suggested that the light Mg isotope preferentially enters into crystallizing carbonates, leading to differentiated carbonatite enriched in the heavy Mg isotope. This was identified in natrocarbonatites at Oldoinyo Lengai which have δ^26^Mg compositions of 0.13–0.37‰^40^. Although the heavy Mg isotope has been demonstrated to be preferentially incorporated into silicate relative to carbonate melts during liquid immiscibility, such fractionation is relatively small (−0.3 to 0.09‰)^40^. Due to the high δ^26^Mg composition of the lower, middle (−0.26 to −0.21‰)^42^ and upper continental crust (−0.52 to 0.92)^43^, crustal contamination will increase the Mg isotope ratio in mantle-derived magmas. The majority of magmatic fractionation processes therefore cannot produce the low levels of δ^26^Mg identified in the carbonatites in this investigation. In contrast, marine dolomite has low δ^26^Mg values of −2.5 to −1‰ over geological time^44^, and its Mg isotopic composition can be preserved during subduction^45^. Therefore, the carbonatite mantle sources in the Qinling orogen may contain recycled sediments.

## Discussion

Several studies indicate that primary mantle-derived carbonatitic melts contain relatively low REE contents^13^,^46^. Experiments have suggested that low REE concentrations in magma are dispersed among the major rock-forming constituents of carbonate minerals, and REE mineralization does not develop^47^. This effect explains why carbonates in most carbonatites have relatively high REE contents^27^,^28^. Statistical data^3^ have shown that there are less than 10 mines with economic REE values among the known 527 carbonatites worldwide. The carbonatites from Qinling (e.g., Miaoya)^39^ and the Himalayan orogenic belts (e.g., Maoniuping and Daluxiang)^48^ host large LREE deposits. Our Mg isotope data indicate that the orogenic carbonatite sources contain sedimentary materials. Modern subducting sediments contain high REE contents^49^, and by analogy this may well have been a characteristic of ancient subducted sediments. Experimental investigations have shown that melting of carbonated eclogite or pelite can form a carbonate melt^50^,^51^. Geochemical evidence also shows that the Tromsø Nappe carbonatites in the Caledonian orogenic belt have an genetic relationship with associated eclogite^52^,^53^. These results imply that recycled sediments are a potential REE source for carbonatite magmas. However, the NQ carbonatites are also enriched in HREE and Mo, thus distinguishing them from the other orogenic carbonatites with LREE enrichment and Mo depletion^39^,^48^. Experimental studies suggest that REE and Mo do not preferentially partition into carbonatite-exsolved fluids^54^, and geochronological data are not consistent with their derivation from later igneous sources within the Qinling region. Therefore, post-magmatic fluid metasomatism cannot explain HREE and Mo enrichment in the NQ carbonatites. This indicates that the primary carbonatitic magmas may have contained high HREE and Mo concentrations, and have undergone a different evolution in the orogen.

**Figure 1:**
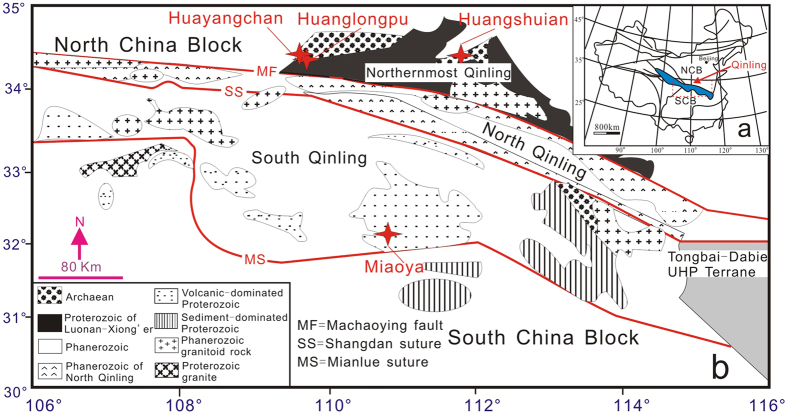
The location of carbonatites in the Qinling orogen and their geological setting. (**a**) Simplified map showing the location of the Qinling orogenic belt, amalgamation of the North China Block (NCB) and South China Block (SCB) along the Qinling, modified after ref. 18. (**b**) Sketch geological map of the carbonatites in Qinling orogenic belt, modified after ref. 18.

**Figure 2:**
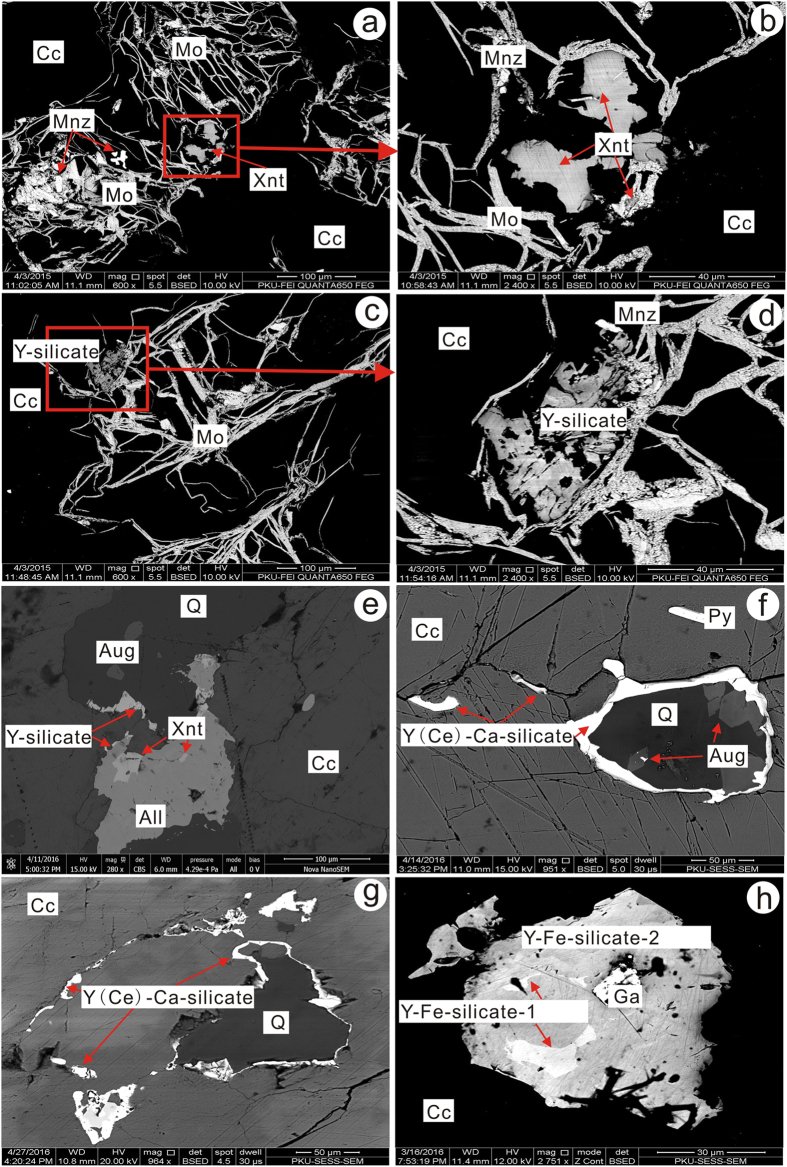
Representative back scatter electron images of REE minerals. All, allanite-(Ce); Aug, augite; Cc, calcite; Ga, galena; Mo, molybdenite; Mnz, monazite-(Ce); Py, pyrite; Q, quartz; Xnt, xenotime-(Y); Y-silicate, Y(Ce)-Ca-silicate and Y-Fe-silicate, unidentified HREE-bearing silicate minerals.

**Figure 3:**
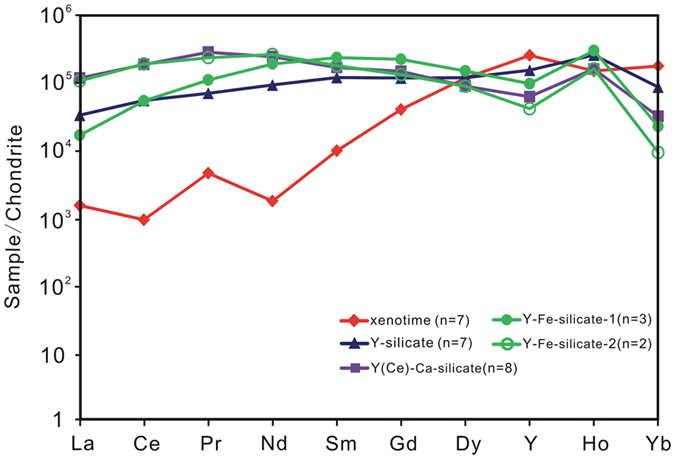


**Figure 4:**
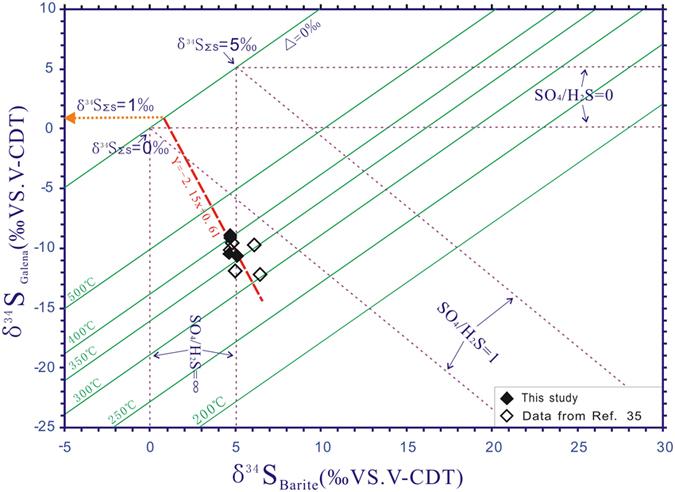
Plots of δ^34^S_galena_ vs. δ^34^S_barite_ values for sulfide and sulfate mineral assemblages from the NQ carbonatite. The straight line of negative slope represents a co-variation in δ^34^S_sulfide_ and δ^34^S_sulfate_ values generated through SO_4_^2−^-H_2_S isotope exchange over a wide range of temperatures^33^. The absolute value (2.15) of the slope of the straight line is equivalent to the molar ratio of SO_4_/H_2_S in the liquids and the sulfur isotopic equilibrium exchange temperatures are between 290 °C and 420 °C. The δ^34^S_ΣS_ value of the carbonatitic liquids is 1‰, estimated from the intersection of the straight line with the line of unit slope which is characterized by non sulfide-sulfate fractionation (Δ = 0‰)., data from ref. 70.

**Figure 5:**
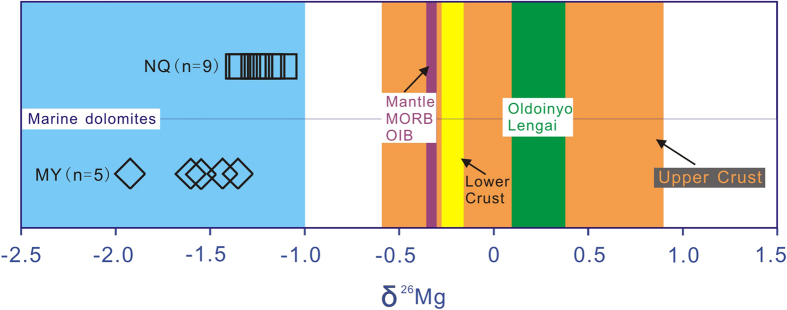
Mg isotopic compositions of NQ and Miaoya (MY) carbonatites in northernmost and southernmost Qinling orogen. Coloured bars show the reference compositions of mantle peridotites^37^, MORB^37^, OIB^37^, upper^43^ and lower crusts^42^, and Oldoinyo Lengai rifting-carbonatites^40^, featuring with distinct Mg isotopic compositions, which are higher than those of the orogenic carbonatites. The carbonatites have similar δ^26^Mg values to marine dolomites^44^.

The Late Triassic carbonatites from Miaoya in the southernmost Qinling orogen are compared with the NQ rocks in Fig. 1b. The Miaoya carbonatites occur as stocks close to the collisional suture between the South and North China Blocks, and they are inferred to have formed from melting of subducted oceanic crust^18^. The rocks contain economic LREE ores and lack HREE minerals and molybdenite. They have a slightly older monazite age of 233 Ma^18^ compared to the NQ monazite (209–214 Ma) and molybdenite (221 Ma) ages. Although the NQ and Miaoya carbonatites have similar light Mg isotope compositions, they show obvious differences in their initial Sr-Nd isotope systematics. The Miaoya carbonatites (initial ^87^Sr/^86^Sr = 0.7036–0.7040 and ε_Nd_ = 0.6 to −1.1)^18^ fall into the HIMU-EM1 trend, corresponding to most young carbonatites worldwide^55^. By contrast, the NQ carbonatites have more radiogenic Sr (initial ^87^Sr/^86^Sr = 0.705–0.706) and lower Nd isotopes ratios (ε_Nd_ = −10.1 to −4.3)^31^. These differences can hardly be attributed to mantle heterogeneity within a relatively confined area.

The lower crust of the north part of the Qinling has higher Mo content (2.04 ppm) than that in the South Qinling, other major tectonic units in eastern China (0.52–0.55 ppm)^56^ and average lower crust worldwide (0.6 ppm)^57^, and is an important Mo source. On this basis we propose the following model for the origin of HREE-Mo rich carbonatites in such an orogenic setting. The formation of both the Late Triassic NQ and Miaoya carbonatites followed the closure of the Mianlue ocean and collision of the South and North China Blocks^18^. Subducted carbonate-bearing ocean crust melted to form carbonatite magmas which intruded in a post-collisional setting. The NQ carbonatites were then emplaced about 10 Ma later than the Miaoya rocks, and they underwent a relatively longer residence time in the mantle source. There are two possible mechanisms for the NQ carbonatite magma interaction with lower crustal materials. During the inter-continental collision, the lower continental crust thickened to 37–82 km^58^, resulting in transformation of the mafic underplate to eclogite. The P-T conditions at the depth of eclogitic crust could then stabilize the carbonate melt (>2 GPa)^9^,^12^. The thickened lower crust may have been directly metasomatized by the upwelling carbonate melt. Alternatively, the eclogitized crust had a higher density than the sub-continental lithospheric mantle which resulted in the delamination of lower crust into the carbonatite mantle source. Breakdown of HREE-enriched eclogitic garnets would then release the HREE into the carbonatite melts. A similar reaction was proposed based on the observation of garnet in peridotite xenoliths^59^, which shows that the metasomatized garnets have lower HREE-contents than the unmetasomatized ones.

The lower crust, when compared to mantle assemblages, is relatively rich in silicon and reactions therefore would have increased silica contents in the reacted carbonatitic melts. The presence of primary HREE-silicate minerals in the NQ carbonatites implies that silicon may play a role in HREE enrichment. Experimental results suggest that increased Si content in carbonatitic melts favors REE incorporation into apatite, particularly HREE (e.g., D^La^_apatite/melt_ = 1.36–1.43, D^Yb^_apatite/melt_ = 2.96–3.06)^60^. Apatite is a major REE carrier in silica-rich systems, incorporating REE via the mechanism [Ca^2+^]_−1_[P^+5^]_−1_[REE^3+^][Si^4+^]^61^, whereas all REE are incompatible with respect to apatite in a silica-poor system (D_REE_^apatite/melt^ < 1)^62^. A study on an REE-rich syenite in Quebec, Canada, showed that HREE will preferentially partition into an immiscible Fe-Si-rich magma relative to the coeval syenite magma^63^.

In conclusion, the HREE-Mo-rich carbonatites in the NQ can be inferred to represent mixtures between carbonatite melt and a component from an eclogitic lower crust. High Mo concentrations have been determined in the lower crust from our study area^56^. Eclogite xenoliths in Mesozoic adakitic porphyries from the southeastern margin of the North China Block^64^ document the existence of an eclogitic crust within the deep lithosphere (>1.5 GPa) in the NQ. The eclogite yields a Sm-Nd whole-rock–garnet isochron and zircon U-Pb dates of 220 Ma^64^. Carbonatites associated with eclogite have also been observed in the orogenic belt of the northern Scandinavian Caledonides^52^,^53^. Although it is accepted that carbonatite metasomatism plays an important role in the modification of the chemistry of the lithospheric mantle^65^, it is not yet known if carbonatite melt generation involves metasomatism of thickened lower crust in orogenic belts. This process may have had the potential to result in HREE enrichment in the carbonatite magma. Apart from the Huayangchuan and Huanglongpu occurrences in this study, another Late Triassic carbonatite (molybdenite Re-Os age of 210 Ma) from Huangshuian^66^ in the middle NQ (Fig. 1) has been recently discovered, thus increasing the geographic extent of these geochemically anomalous carbonatites. Thus, overall, the NQ orogen has promising potential as a REE-mineralized belt.

## Methods

### Monazite U-Th-Pb dating

U-Th-Pb analyses of the monazite were performed using the Cameca IMS-1280 (SIMS) at the Institute of Geology and Geophysics, Chinese Academy of Sciences (CAS). The O^2−^ primary ion beam was accelerated at 13 kV with an intensity of ca. 2–3 nA. The aperture illumination mode (Kohler illumination) was used with a 200 μm primary beam mass filter (PBMF) aperture to produce even sputtering over the entire analyzed area. The ellipsoidal spot is about 20 × 30 μm in size. Positive secondary ions were extracted with a 10 kV potential. Sample charging effects were minimized by optimizing the energy offset to maximum transmission in a 60 eV energy window at the start of each analysis, using Th^+^ as reference peak. A mass resolution of ca. 5,500 (defined at 1% peak height) was used, which is enough to separate U, Th, and Pb isotopes from isobaric interferences, such as REE oxides, except for ^204^Pb which could be interfered by ThNdO_2_^+^. Monazite 44069 was used as a standard.

### EPMA and LA-ICPMS analysis of minerals

The major element compositions of the HREE minerals in the carbonatites were determined using an EPMA-JXA-8230 electron microprobe with wavelength-dispersive X-ray spectrometry (WDS) at the Key Laboratory of Metallogeny and Mineral Assessment, Chinese Academy of Geological Sciences. The measurement conditions were 15 kV accelerating voltage and 20 nA beam current, with an electron beam of 2–5 μm diameter. According to the components of separated minerals, their own set of appropriate matrix-specific standards (both synthetic and natural) and optimal instrumental conditions (detector type, counting statistics and beam settings) were carefully chosen by performing multiple measurements. All data were corrected with standard ZAF correction procedures. The raw WDS data of REE were corrected on the basis of empirical interference values for REE using well-characterized REE glasses, synthetic standards and orthophosphates.

*In-situ* laser-ablation (LA) ICP-MS analyses of fluorapatites were performed using an Agilent 7500Ce mass-spectrometer coupled to a 193-nm ArF excimer laser at the Peking University. The diameter of the ablation spot used was 30 μm. NIST 610 glass was used as a calibration standard for all samples. The element used for the internal standard was Ca, measured as ^43^Ca and expressed as CaO, which was independently measured by electron microprobe. The analytical error is within 5%.

### Fluid inclusion measurement

Laser Raman spectroscopic analyses of compositions of the individual fluid inclusions in the carbonatites were performed using the LabRam HR800 at the Institute of Geology and Geophysics, CAS. The laser source was an argon ion laser with wavelength of 532 nm and output power of 44 mV. Count time for each measured spectrum is 3 s and counted once for each cm^−1^. The spectral resolution was 0.65 cm^−1^ with a beam size of 1 μm. The peak was taken from 100 to 4000 cm^−1^ once in full-wave band. Instrumental settings were kept constant during all the analyses. Solid minerals in opened inclusions were identified by LEO-1450vp scanning electron microscope with an attached Oxford INCA-ENERGY 300 X-ray spectrometer operated at 15 kV at the same Institute.

The composition of fluid inclusions in the carbonatitic calcite was measured by LA-ICPMS at the Advanced Analytical Centre, James Cook University, Australia. A GeoLas 193 nm ArF excimer laser coupled with a Varian 820 quadrupole ICP mass spectrometer with He as the carrier gas in the ablation chamber was used. To obtain maximum signal sensitivities, a Y-piece was used to admix Ar prior to introduction to the ICP. The laser beam diameter ranged between 24 and 44 μm, depending on the inclusion size and depth within the sample. Chlorine and Br concentrations were quantified using the scapolite BB1 standard^67^, whereas all other elements were quantified using NIST 610 glass. Sodium concentrations, derived from microthermometry, were used as the internal standard. The SILLS software was used to perform all data reduction procedures.

### Sulfur isotope analysis

Sulfur isotopic compositions of barite and sulfides from the carbonatites were measured at the Institute of Geochemistry, CAS, using a continuous-flow isotope ratio mass spectrometer (IsoPrime). Reproducibilities are ± 0.2‰, and the results are expressed conventionally as per mil (‰) variation relative to V-CDT.

### Mg isotope analysis

The Mg isotopic pretreatment has been described by ref. 68. The total procedural blank for Mg was <15 ng. The isotope was analysed using a Neptune MC-ICP-MS at the China University of Geosciences (Beijing). The analytical results are expressed as δ-notation in per mil relative to DSM3: δ^X^Mg = [(^X^Mg/^24^Mg)_sample_/(^X^Mg/^24^Mg)_DSM3_ − 1] × 1000, where X refers to mass 25 or 26 and DSM3 is a magnesium solution made from pure Mg metal. The analyses of standard BCR-2, BHVO-2, Kilbourne Hole olivine, and Hawaii seawater were in good agreement with recommended values within 2σ error^69^.

## Additional Information

**How to cite this article**: Song, W. *et al*. Origin of unusual HREE-Mo-rich carbonatites in the Qinling orogen, China. *Sci. Rep.* **6**, 37377; doi: 10.1038/srep37377 (2016).

**Publisher's note**: Springer Nature remains neutral with regard to jurisdictional claims in published maps and institutional affiliations.

## Supplementary Material

Supplemntary Figures S1ߝS5 and Tables S1ߝS5

## Figures and Tables

**Table 1:** Mg isotope compositions (‰) of carbonatites in Qinling orogen.

Sample	δ^25^Mg	2 SD	δ^26^Mg	2 SD
NQ-1	−0.68	0.02	−1.23	0.06
NQ-2	−0.59	0.02	−1.11	0.05
NQ-3	−0.67	0.02	−1.28	0.06
NQ-4	−0.65	0.01	−1.21	0.05
NQ-5	−0.67	0.04	−1.25	0.04
NQ-6	−0.56	0.01	−1.07	0.03
NQ-7	−0.67	0.02	−1.27	0.07
NQ-8	−0.67	0.05	−1.23	0.07
NQ-9	−0.67	0.03	−1.24	0.03
MY-1	−0.75	0.04	−1.44	0.06
MY-2	−1.03	0.04	−1.89	0.04
MY-3	−0.76	0.01	−1.37	0.03
MY-4	−0.81	0.03	−1.53	0.08
MY-5	−0.85	0.05	−1.58	0.07

NQ, Huayangchuan and Huanglongpu in northernmost Qinling.

MY, Miaoya in southernmost Qinling.

## References

[b1] United States Geological Survey. Mineral Commodity Summaries 2016, 1–202 (2016).

[b2] Goodenough K. M. . Europe’s rare earth element resource potential: an overview of REE metallogenetic provinces and their geodynamic setting. Ore Geol. Rev. 72, 838–856 (2016).

[b3] Woolley A. R. & Kjarsgaard B. A. Carbonatite occurrences of the world: Map and database. Geological Survey of Canada, Open File 5796 (2008).

[b4] Andersson M. . Carbonatite ring-complexes explained by caldera-style volcanism. Sci. Rep. 3, 1–9 (2013). 10.1038/srep01677 PMC3628075 23591904

[b5] Andersson M. . Magma transport in sheet intrusions of the Alnö carbonatite complex, central Sweden. Sci. Rep. 6, 27635 (2016). 27282420 10.1038/srep27635 PMC4901264

[b6] Wall F., Niku-Paavola V. N., Storey C., Müller A. & Jeffries T. Xenotime-(Y) from carbonatite dykes at Lofdal, Namibia: unusually low LREE: HREE ratio in carbonatite, and the first dating of xenotime overgrowths on zircon. Can. Mineral. 46, 861–877 (2008).

[b7] Xu C., Kynicky J., Chakhmouradian A. R., Qi L. & Song W. A unique Mo deposit associated with carbonatites in the Qinling orogenic belt, central China. Lithos 118, 50–60 (2010).

[b8] Song W. . Genesis of Si-rich carbonatites in Huanglongpu Mo deposit, Lesser Qinling orogen, China and significance for Mo mineralization. Ore Geol. Rev. 64, 756–765 (2015).

[b9] Wallace M. E. & Green D. H. An experimental determination of primary carbonatite magma composition. Nature 335, 343–346 (1988).

[b10] Brooker R. A. & Kjarsgaard B. A. Silicate–carbonate liquid immiscibility and phase relations in the system SiO_2_-Na_2_O-Al_2_O_3_-CaO-CO_2_ at 0.1–2.5 GPa with applications to carbonatite genesis. J. Petrol. 52, 1281–1305 (2011).

[b11] Gittins J. & Jago B. C. Differentiation of natrocarbonatite magma at Oldoinyo Lengai volcano, Tanzania. Mineral. Mag. 62, 759–768 (1998).

[b12] Sweeney R. J. Carbonatite melt compositions in the Earth’s mantle. Earth Planet Sci. Lett. 28, 259–270 (1994).

[b13] Foley S. F. . The composition of near-solidus melts of peridotite in the presence of CO_2_ and H_2_O between 40 and 60 kbar. Lithos 112, 274–283 (2009).

[b14] Walter M. J. . Primary carbonatite melt from deeply subducted oceanic crust. Nature 454, 622–625 (2008). 18668105 10.1038/nature07132

[b15] Veksler I. V. . Partitioning of elements between silicate melt and immiscible fluoride, chloride, carbonate, phosphate and sulfate melts, with implications to the origin of natrocarbonatite. Geochim. Cosmochim. Acta 79, 20–40 (2012).

[b16] Guzmics T., Zajacz Z., Mitchell R. H., Szabó C. & Wälle M. The role of liquid–liquid immiscibility and crystal fractionation in the genesis of carbonatite magmas: insights from Kerimasi melt inclusions. Contrib. Mineral. Petrol. 169, 1–18 (2015).

[b17] Reguir E. P. . Trace-element composition and zoning in clinopyroxene- and amphibole-group minerals: implications for element partitioning and evolution of carbonatites. Lithos 128–131, 27–45 (2012).

[b18] Xu C. . Origin of carbonatites in the south Qinling orogen: implications for crustal recycling and timing of collision between the south and north china blocks. Geochim. Cosmochim. Acta 143, 189–206 (2014).

[b19] Mao J. W. . Tectonic implications from Re-Os dating of Mesozoic molybdenum deposits in the East Qinling-Dabie orogenic belt. Geochim. Cosmochim. Acta 72, 4607–4626 (2008).

[b20] Wall F. & Mariano A. N. Rare earth minerals in carbonatites: a discussion centred in the Kangankunde carbonatite, Malawi, In Rare Earth Minerals: Chemistry, Origin and Ore Deposits, (eds Jones A. P., Wall F., Williams C. T.) 193–225 (Chapman and Hall, 1996).

[b21] Zaitsev A., Demeny A., Sundern S. & Wall F. Burbankite group minerals and their alteration in rare earth carbonatites—source of elements and fluids (evidence from C–O and Sr–Nd isotopica dta). Lithos 62, 15–33 (2002).

[b22] Stein H. J., Markey R. J., Morgan M. J., Du A. & Sun Y. Highly precise and accurate Re-Os ages for molybdenite from the East Qinling-Dabie molybdenum belt, Shaanxi province, China. Econ. Geol. 92, 827–835 (1997).

[b23] Klemm L. M., Pettke T. & Heinrich C. A. Fluid and source magma evolution of the Questa porphyry Mo deposit, New Mexico, USA. Miner. Deposita 43, 533–552 (2008).

[b24] Lentz D. R. Carbonatite genesis: a reexamination of the role of intrusion-related pneumatolytic skarn processes in limestone melting. Geology 27, 335–338 (1999).

[b25] Mitchell R. H. Carbonatites and carbonatites and carbonatites. Can. Mineral. 43, 2049–2068 (2005).

[b26] Meinert L. D., Dipple G. M. & Nicolescu S. World skarn deposits. Econ. Geol. 100th Anniversary Volume, 299–336 (2005).

[b27] Chakhmouradian A. R., Reguir E. P., Couëslan C. & Yang P. Calcite and dolomite in intrusive carbonatites. II. Trace-element variations. Mineral. Petrol. 110, 361–377 (2016).

[b28] Hornig-Kjarsgaard I. Rare earth elements in sovitic carbonatites and their mineral phases. J. Petrol. 39, 2105–2121 (1998).

[b29] Belousova E. A., Griffin W. L., O’Reilly S. Y. & Fisher N. I. Apatite as an indicator mineral for mineral exploration: trace-element compositions and their relationship to host rock type. J. Geochem. Explor. 76, 45–69 (2002).

[b30] Xu C. . Flat rare earth element patterns as an indicator of cumulate processes in the Lesser Qinling carbonatites, China. Lithos 95, 267–278 (2007).

[b31] Xu C. . The origin of enriched mantle beneath North China block: Evidence from young carbonatites. Lithos 127, 1–9 (2011).

[b32] Gomide C. S. . Sulfur isotopes from Brazilian alkaline carbonatite complexes. Chem. Geol. 341, 38–49 (2013).

[b33] Fifarek R. H. & Rye R. O. Stable-isotope geochemistry of the Pierina high-sulfidation Au-Ag deposit, Peru: influence of hydrodynamics on SO_4_^2−^-H_2_S sulfur–isotope exchange in magmatic-steam and steam-heated environments. Chem. Geol. 215, 253–279 (2005).

[b34] Schneider A. The sulfur isotope composition of basaltic rocks. Contrib. Mineral. Petrol. 25, 95–124 (1970).

[b35] Ray J. S., Ramesh R. & Pande K. Carbon isotopes in Kerguelen plume-derived carbonatites: evidence for recycled inorganic carbon. Earth Planet. Sci. Lett. 170, 205–214 (1999).

[b36] Yang W., Teng F. Z., Zhang H. F. & Li S. G. Magnesium isotopic systematics of continental basalts from the North China craton: Implications for tracing subducted carbonate in the mantle. Chem. Geol. 328, 185–194 (2012).

[b37] Teng F. Z. . Magnesium isotopic composition of the Earth and chondrites. Geochim. Cosmochim. Acta 74, 4150–4166 (2010).

[b38] Wang S. J., Teng F. Z., Li S. G. & Hong J. A. Magnesium isotopic systematics of mafic rocks during continental subduction. Geochim. Cosmochim. Acta 143, 34–48 (2014).

[b39] Xu C., Kynicky J., Chakhmouradian A. R. Campbell I. H. & Charlotte M. A. Trace-element modeling of the magmatic evolution of rare-earth-rich carbonatite from the Miaoya deposit, central china. Lithos 118, 145–155 (2010).

[b40] Li W. Y. . Magnesium isotope fractionation during carbonatite magmatism at OldoinyoLengai, Tanzania. Earth Planet. Sci. Lett. 444, 26–33 (2016).

[b41] Macris C. A., Young E. D. & Manning C. E. Experimental determination of equilibrium magnesium isotope fractionation between spinel, forsterite, and magnesite from 600 to 800 °C. Geochim. Cosmochim. Acta 118, 18–32 (2013).

[b42] Yang W. . Magnesium isotopic composition of the deep continental crust. Am. Mineral. 101, 243–252 (2016).

[b43] Li W. Y. . Heterogeneous magnesium isotopic composition of the upper continental crust. Geochim. Cosmochim. Acta 74, 6867–6884 (2010).

[b44] Huang K. J. . Magnesium isotopic compositions of the Mesoproterozoic dolostones: Implications for Mg isotopic systematics of marine carbonates. Geochim. Cosmochim. Acta 164, 333–351 (2015).

[b45] Wang S. J., Teng F. Z. & Li S. G. Tracing carbonate-silicate interaction during subduction using magnesium and oxygen isotopes. Nat. Commun. 5, 5328 (2014). 25367068 10.1038/ncomms6328

[b46] Ionov D. A. & Harmer R. E. Trace element distribution on calcite-dolomite carbonatites from Spitskop: inferences for differentiation of carbonatite magmas and the origin of carbonates in mantle xenoliths. Earth Planet. Sci. Lett. 198, 495–510 (2002).

[b47] Wyllie P. J., Jones A. P. & Deng J. Rare earth elements in carbonate-rich melts from mantel to crust, In Rare Earth Minerals: Chemistry, Origin and Ore Deposits, (eds Jones A. P., Wall F., Williams C. T.) 77–103 (Chapman and Hall, 1996).

[b48] Hou Z., Liu Y., Tian S., Yang Z. & Xie Y. Formation of carbonatite-related giant rare-earth-element deposits by the recycling of marine sediments. Sci. Rep. 5, 10231 (2015). 26035414 10.1038/srep10231 PMC4451788

[b49] Plank T. & Langmuir C. H. The chemical composition of subducting sediment and its consequences for the crust and mantle. Chem. Geol. 145, 325–394 (1998).

[b50] Yaxley G. M. & Brey G. P. Phase relations of carbonate-bearing eclogite assemblages from 2.5 to 5.5 GPa: Implications for petrogenesis of carbonatites. Contrib. Mineral. Petrol. 146, 606–619 (2004).

[b51] Grassi D. & Schmidt M. W. The melting of carbonated pelites from 70 to 700 km depth. J. Petrol. 52, 765–789 (2011).

[b52] Ravna E., Kullerud K., Davidsen B. & Selbekk R. S. Peculiar carbonate-rich HP/UHP rocks from the Tromsø Nappe - metacarbonatite or remobilized marble? AGU Fall Conf. Abstr. V41C-0719 (AGU General Assembly, 2007).

[b53] Ravna E., Zozulya D. R., Kullerud K. & Serov P. Sr-Nd isotope data for carbonatite and related UHP rocks from Tromsø Nappe, Northern Scandinavian Caledonides. Alkaline Magmatism and Related Strategic Metal Deposits 2015 Conf. Abstr. (Russian Kola Science Centre, 2015).

[b54] Song W., Xu C., Veksler I. V. & Kynicky J. Experimental study of REE, Ba, Sr, Mo and W partitioning between carbonatitic melt and aqueous fluid with implications for rare metal mineralization. Contrib. Mineral. Petrol. 171, 1–12 (2016).

[b55] Bell K. & Simonetti A. Source of parental melts to carbonatites-critical isotopic constraints. Mineral. Petrol. 98, 77–89 (2010).

[b56] Gao S. . Chemical composition of the continental crust as revealed by studies in East China. Geochim. Cosmochim. Acta 62, 1959–1975 (1998).

[b57] Wedepohl K. H. The composition of the continental crust. Geochim. Cosmochim. Acta 59, 1217–1232 (1995).

[b58] Gao S. . How mafic is the lower continental crust? Earth Planet. Sci. Lett. 161, 101–117 (1998).

[b59] Achterbergh E. V., Griffin W. L. & Stiefenhofer J. Metasomatism in mantle xenoliths from the Letlhakane kimberlites: estimation of element fluxes. Contrib. Mineral. Petrol. 141, 397–414 (2001).

[b60] Hammouda T., Chantel J. & Devidal J. L. Apatite solubility in carbonatitic liquids and trace element partitioning between apatite and carbonatite at high pressure. Geochim. Cosmochim. Acta 74, 7220–7235 (2010).

[b61] Pan Y. & Fleet M. E. Compositions of the apatite-group minerals: substitution mechanisms and controlling factors. Rev. Min. Geochem. 48, 13–49 (2002).

[b62] Klemme S. & Dalpé C. Trace-element partitioning between apatite and carbonatite melt. Am. Mineral. 88, 639–646 (2003).

[b63] Petrella L., Williams-Jones A. E., Goutier J. & Walsh J. The nature and origin of the rare earth element mineralization in the Misery syenitic intrusion, northern Quebec, Canada. Econ. Geol. 109, 1643–1666 (2014).

[b64] Xu W. L., Gao S., Wang Q. H., Wang D. Y. & Liu Y. S. Mesozoic crustal thickening of the eastern North China craton: Evidence from eclogite xenoliths and petrologic implications. Geology 34, 721–724 (2006).

[b65] Green D. H. & Wallace M. E. Mantle metasomatism by ephemeral carbonatite melts. Nature 336, 459–462 (1988).

[b66] Cao J. . Geological characteristics and molybdenite Re-Os isotopic dating of Huangshuian carbonatite vein-type Mo (Pb) deposit in Songxian County, Henan Province. Mineral Deposits 33, 53–69 (2014).

[b67] Kendrick M. A. High precision Cl, Br and I determinations in mineral standards using the noble gas method. Chem. Geol. 292, 116–126 (2013).

[b68] Shen B., Wimpenny J., Lee C. T. A., Tollstrup D. & Yin Q. Z. Magnesium isotope systematics of endoskarns: Implications for wallrock reaction in magma chambers. Chem. Geol. 356, 209–214 (2013).

[b69] Teng F. Z. . Magnesium isotopic compositions of international geostandards. Geostand. Geoanal. Res. 39, 329–339 (2015).

[b70] Huang D. H., Wang Y. C., Nie F. J. & Jiang X. J. Isotopic composition of sulfur, carbon and oxygen and source material of the Huanglongpu carbonatite dyke-type of molybdenum deposits. Acta Geol. Sin. 3, 252–264 (1984).

