# Supplementary material for: Origin of unusual HREE-Mo-rich carbonatites in the Qinling orogen, China
Source: Sci Rep. 2016 Nov 18;6:37377. doi: 10.1038/srep37377 (PMC5114661; doi:10.1038/srep37377)
Supplement: Supplemntary Figures S1ߝS5 and Tables S1ߝS5 [file srep37377-s1.doc]

# Supplementary Information

# Origin of unusual HREE-Mo-rich carbonatites in the Qinling orogen, China

Authors: Wenlei Song^1^, Cheng Xu^1^, Martin P. Smith^2^, Jindrich Kynicky^3^, Kangjun Huang^1^, Chunwan Wei^1^, Li Zhou^4^, and Qihai Shu^5^

1: Laboratory of Orogenic Belts and Crustal Evolution, School of Earth and Space Sciences, Peking University, Beijing 100871, China

2: School of Environment and Technology, University of Brighton, Brighton BN41 2HQ, United Kingdom

3: Department of Geology and Pedology, Mendel University, Brno 61300, Czech Republic

4: Key Laboratory of High-temperature and High-pressure Study of the Earth’s Interior, Institute of Geochemistry, Chinese Academy of Sciences, Guiyang 550002, China

5: State Key Laboratory of Geological Processes and Mineral Resources, China University of Geosciences, Beijing 100083, China

Email address of the corresponding author (C. Xu): xucheng1999@pku.edu.cn

**This file includes Supplementary Figures S1 to S5 and Table S1 to S5.**

# Supplementary Figures


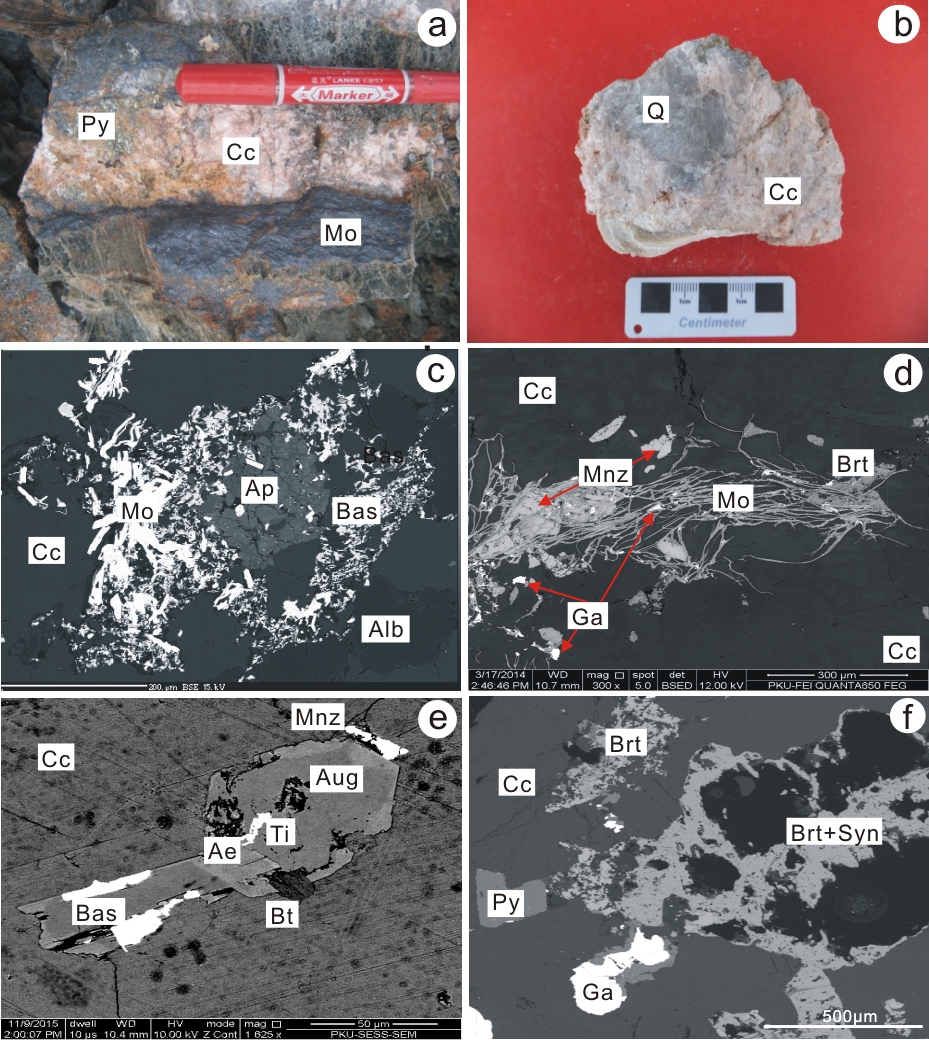


Supplementary Figure S1 **|** Field specimens of carbonatites (a, b) and back-scattered electron images of their mineral compositions (c-f). Ae, aegirine; Ap, fluorapatite; Alb, plagioclase; Aug, augite; Bas, bastnäsite-(Ce); Brt, barite; Bt, biotite; Cc, calcite; Ga, galena; Mo, molybdenite; Mnz, monazite-(Ce); Syn, synchysite-(Ce); Py, pyrite; Q, quartz; Ti, titanite.


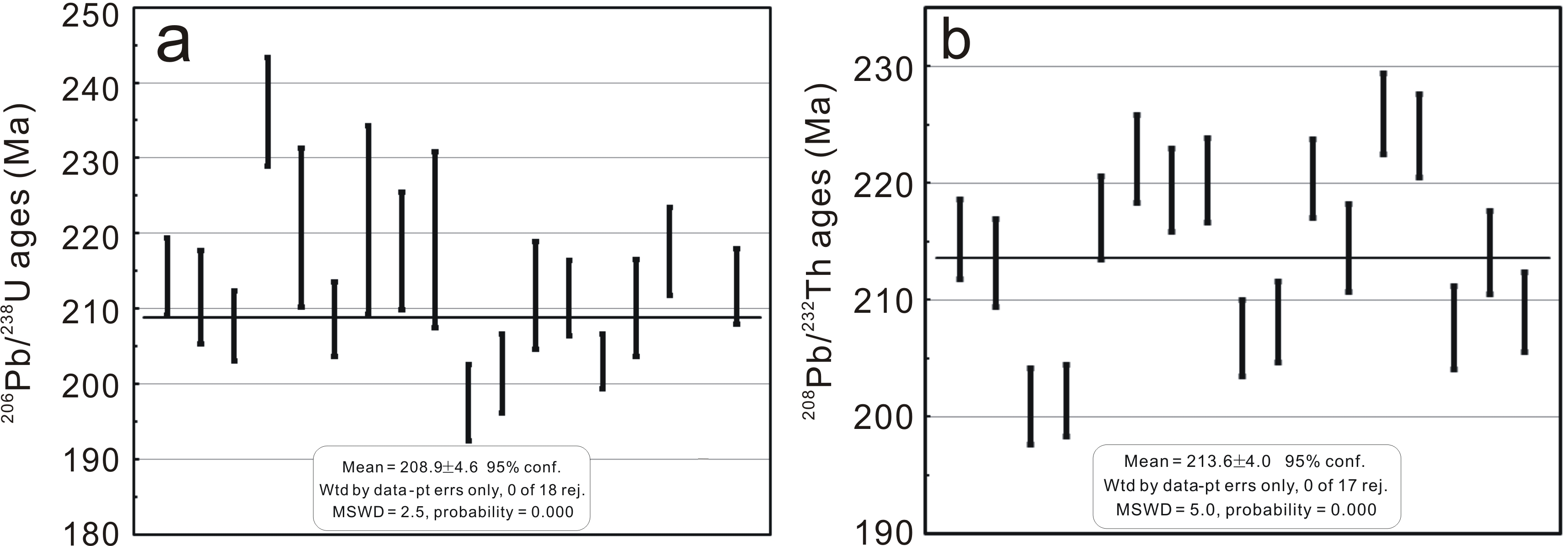


Supplementary Figure S2 | Weighted average plots of (a) ^206^Pb/^238^U and (b) ^208^Pb/^232^Th ages of monazites.


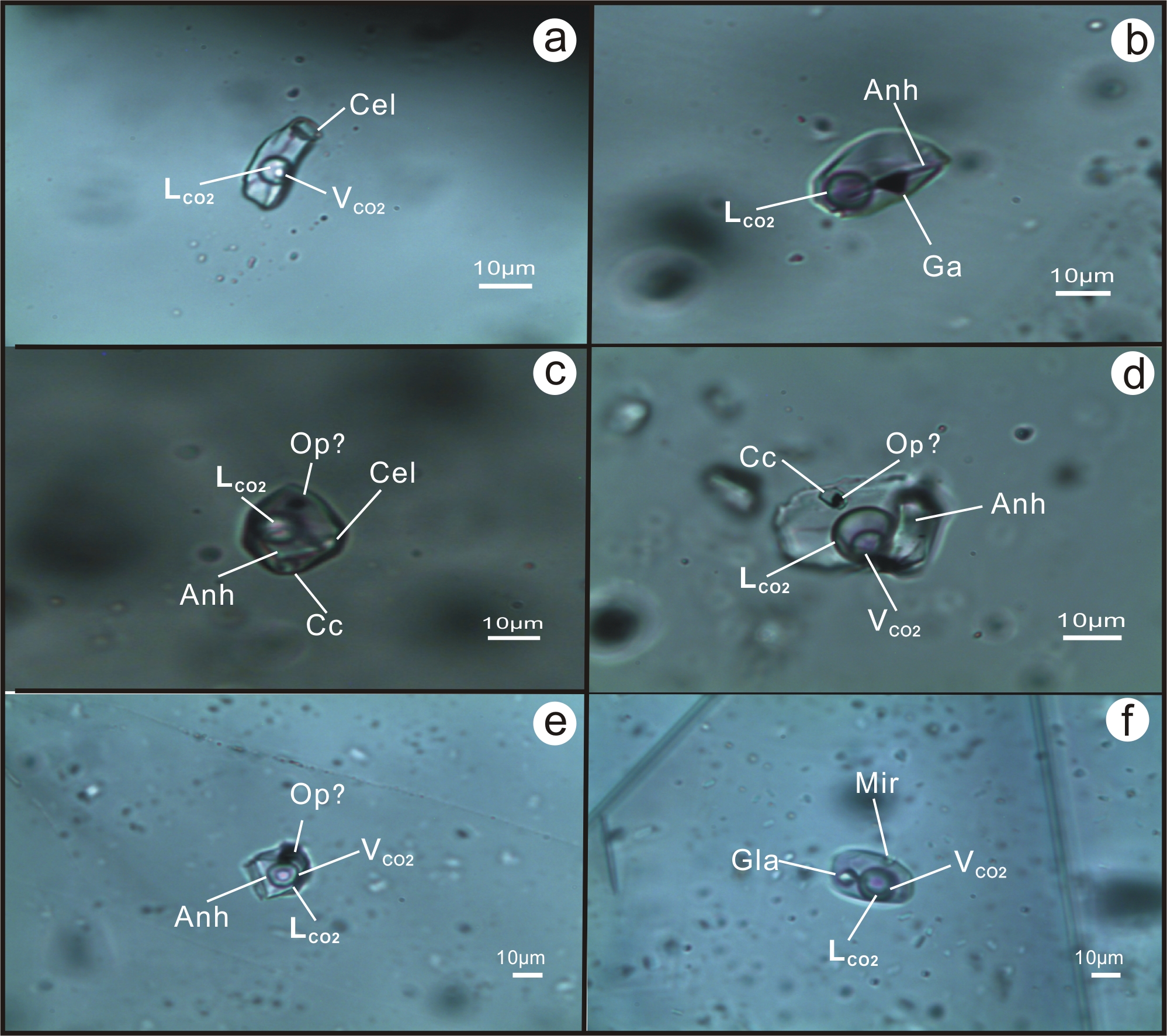


Supplementary Figure S3 | Photomicrographs of solid-bearing fluid inclusions in quartz (a-d) and calcite (e, f) from the NQ carbonatites. V_CO2_, vapor CO_2_; L_CO2_, liquid CO_2_; Cel, celestine; Anh, anhydrite; Ga, galena; Cc, calcite; Gla, glaserite; Mir, mirabilite; OP?, unidentified opaque daughter mineral; Tr?, unidentified transparent daughter mineral.


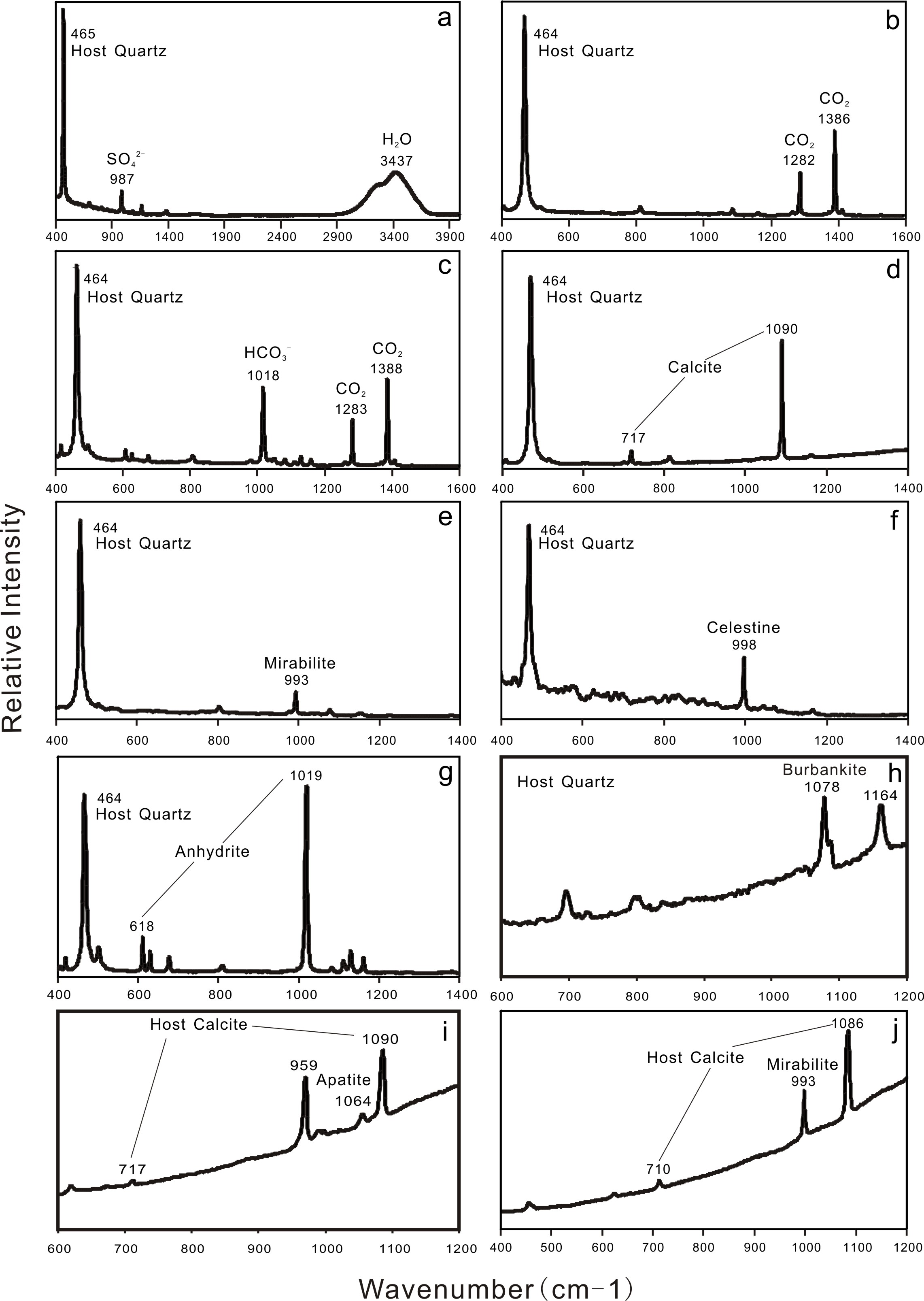


Supplementary Figure S4 | Raman spectrum of liquid and solid phases in fluid inclusions hosted in quartz (a-h) and calcite (i-g) from the carbonatites. (a) SO_4_^2−^ in the aqueous phase of the aqueous A-type fluid inclusion in quartz; (b) CO_2_ in the pure carbonic (PC) fluid inclusion; (c) HCO_3_^-^ in the liquid CO_2_ phase of the aqueous-carbonic (AC) fluid inclusion in quartz; (d-h) Calcite, mirabilite, celestine, anhydrite and burbankite daughter minerals in the solid-bearing aqueous-carbonic (MS) fluid inclusion in quartz, respectively; (i-j) Apatite and mirabilite daughter minerals in the solid-bearing aqueous-carbonic (MS) fluid inclusion in calcite, respectively.


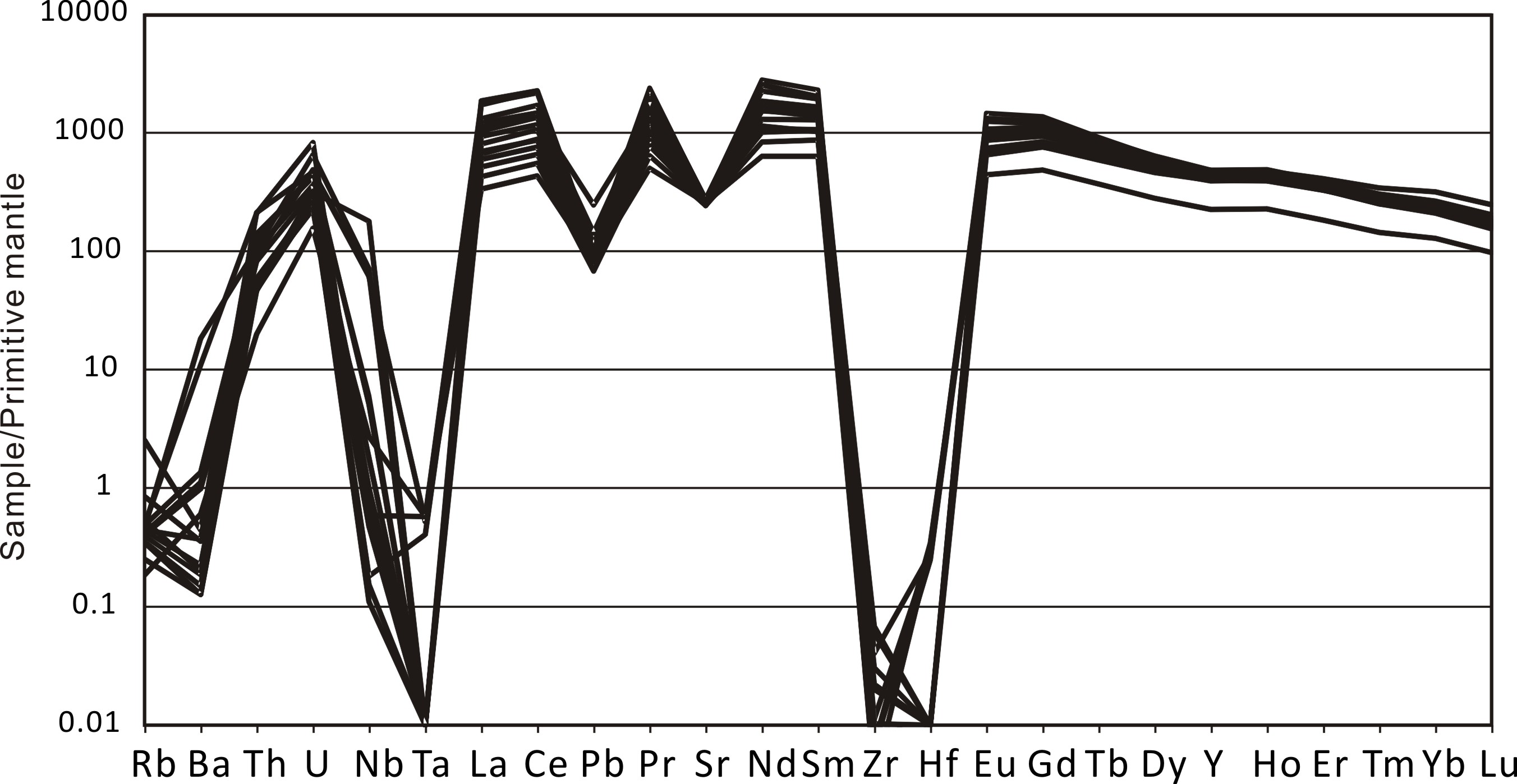


Supplementary Figure S5 | Primitive mantle normalized trace elements of fluorapatites in the carbonatites.

### Supplementary Tables

Supplementary Table S1 | Compositions (wt.%) of HREE minerals in northernmost Qinling carbonatites

|  |  | |  |  |  |  |  |  | | | | |  |  |
| --- | --- | --- | --- | --- | --- | --- | --- | --- | --- | --- | --- | --- | --- | --- |
|  | Xenotime-(Y) | |  |  |  |  |  | Y-silicate | | | | |  |  |
| F | 0.07 | 0.05 | 0.04 | 0.05 | 0.03 | 0.06 | 0.03 | 0.01 | b.d | 0.02 | 0.02 | 0.01 | b.d | b.d |
| MgO | n.a | n.a | n.a | n.a | n.a | n.a | n.a | n.a | n.a | n.a | n.a | n.a | n.a | n.a |
| K_2_O | n.a | n.a | n.a | n.a | n.a | n.a | n.a | n.a | n.a | n.a | n.a | n.a | n.a | n.a |
| Na_2_O | n.a | n.a | n.a | n.a | n.a | n.a | n.a | n.a | n.a | n.a | n.a | n.a | n.a | n.a |
| FeO | n.a | n.a | n.a | n.a | n.a | n.a | n.a | n.a | n.a | n.a | n.a | n.a | n.a | n.a |
| SiO_2_ | 0.14 | 0.34 | 0.04 | 0.13 | 0.05 | 0.20 | 0.22 | 28.25 | 27.5 | 24.54 | 24.15 | 27.05 | 26.16 | 26.90 |
| CaO | 0.07 | 0.14 | 0.05 | 0.05 | 0.05 | 0.09 | 0.08 | 1.87 | 0.24 | 0.28 | 0.19 | 3.19 | 0.17 | 2.85 |
| MnO | n.a | n.a | n.a | n.a | n.a | n.a | n.a | n.a | n.a | n.a | n.a | n.a | n.a | n.a |
| Al_2_O_3_ | n.a | n.a | n.a | n.a | n.a | n.a | n.a | n.a | n.a | n.a | n.a | n.a | n.a | n.a |
| ThO_2_ | 0.01 | 0.24 | 0.08 | 0.18 | 0.07 | 0.04 | 0.15 | 0.03 | b.d | 0.07 | b.d | 0.02 | 0.05 | 0.07 |
| UO_2_ | 0.07 | 0.19 | 0.06 | 0.08 | 0.08 | 0.01 | 0.28 | b.d | b.d | b.d | 0.04 | 0.04 | b.d | 0.08 |
| PbO | 0.44 | 0.44 | 0.33 | 0.26 | 0.32 | 0.48 | 0.52 | 0.08 | 0.02 | 0.21 | 0.04 | 0.01 | b.d | b.d |
| Y_2_O_3_ | 50.43 | 49.85 | 50.92 | 51.56 | 52.23 | 52.31 | 49.43 | 31.88 | 31.75 | 33.52 | 30.1 | 30.19 | 28.21 | 29.17 |
| La_2_O_3_ | b.d | 0.04 | 0.01 | 0.02 | 0.12 | b.d | b.d | 0.72 | 1.07 | 0.80 | 1.06 | 0.60 | 1.50 | 0.94 |
| Ce_2_O_3_ | b.d | 0.06 | 0.21 | b.d | 0.03 | 0.05 | 0 | 3.46 | 4.50 | 3.42 | 4.08 | 2.93 | 5.69 | 3.90 |
| Pr_2_O_3_ | 0.1 | b.d | 0.01 | b.d | b.d | 0.04 | 0.06 | 0.57 | 0.87 | 0.62 | 0.96 | 0.63 | 1.14 | 0.83 |
| Nd_2_O_3_ | 0.08 | 0.19 | b.d | 0.2 | b.d | 0.07 | 0.03 | 3.43 | 4.93 | 4.14 | 5.27 | 4.56 | 7.18 | 5.52 |
| Sm_2_O_3_ | 0.32 | 0.3 | 0.13 | 0.15 | 0.1 | 0.18 | 0.11 | 1.46 | 1.64 | 1.55 | 2.72 | 2.28 | 2.46 | 2.69 |
| Eu_2_O_3_ | b.d | b.d | b.d | b.d | b.d | b.d | b.d | 0.14 | 0.75 | 0.21 | 0.17 | 0.04 | 1.03 | b.d |
| Gd_2_O_3_ | 1.43 | 1.47 | 0.74 | 0.97 | 0.81 | 0.69 | 0.85 | 2.38 | 2.02 | 2.37 | 3.46 | 3.18 | 2.61 | 3.49 |
| Dy_2_O_3_ | 3.49 | 3.39 | 3.62 | 3.42 | 3.51 | 3.61 | 3.48 | 4.24 | 3.18 | 3.71 | 3.67 | 3.49 | 3.02 | 3.46 |
| Ho_2_O_3_ | 1.13 | 1.25 | 0.93 | 0.97 | 0.92 | 0.9 | 0.88 | 1.73 | 1.38 | 1.42 | 2.09 | 1.76 | 1.45 | 1.98 |
| Er_2_O_3_ | 2.66 | 2.07 | 2.90 | 2.91 | 2.94 | 3.09 | 2.98 | 1.65 | 1.58 | 1.57 | 1.30 | 1.71 | 1.19 | 1.40 |
| Tm_2_O_3_ | n.a | n.a | n.a | n.a | n.a | n.a | n.a | n.a | n.a | n.a | n.a | n.a | n.a | n.a |
| Yb_2_O_3_ | 3.18 | 2.85 | 3.46 | 3.97 | 2.70 | 3.12 | 3.69 | 1.78 | 1.81 | 1.82 | 1.34 | 1.97 | 1.32 | 1.59 |
| Lu_2_O_3_ | n.a | n.a | n.a | n.a | n.a | n.a | n.a | n.a | n.a | n.a | n.a | n.a | n.a | n.a |
| P_2_O_5_ | 34.91 | 35.42 | 35.91 | 34.83 | 35.57 | 34.78 | 35.77 | b.d | b.d | b.d | b.d | b.d | b.d | b.d |
| SO_3_ | n.a | n.a | n.a | n.a | n.a | n.a | n.a | n.a | n.a | n.a | n.a | n.a | n.a | n.a |
| Cl | n.a | n.a | n.a | n.a | n.a | n.a | n.a | n.a | n.a | n.a | n.a | n.a | n.a | n.a |
| F=O | -0.03 | -0.02 | -0.02 | -0.02 | -0.01 | -0.03 | -0.01 |  |  | -0.01 | -0.01 |  |  |  |
| Total | 98.48 | 98.28 | 99.44 | 99.72 | 99.51 | 99.67 | 98.56 | 83.68 | 83.25 | 80.24 | 80.62 | 83.67 | 83.20 | 84.86 |
|  | *Cations per 4O* | | | | | | | *Cations per 7O* | | | | | | |
| F | 0.007 | 0.005 | 0.004 | 0.005 | 0.003 | 0.006 | 0.000 | 0.002 | 0.000 | 0.005 | 0.005 | 0.002 | 0.000 | 0.000 |
| Si | 0.005 | 0.011 | 0.001 | 0.004 | 0.002 | 0.007 | 0.007 | 2.085 | 2.076 | 1.971 | 1.969 | 2.029 | 2.039 | 2.017 |
| Ca | 0.002 | 0.005 | 0.002 | 0.002 | 0.002 | 0.003 | 0.003 | 0.148 | 0.019 | 0.024 | 0.017 | 0.256 | 0.014 | 0.229 |
| Th | 0.000 | 0.002 | 0.001 | 0.001 | 0.001 | 0.000 | 0.001 | 0.001 | 0.000 | 0.001 | 0.000 | 0.000 | 0.001 | 0.001 |
| U | 0.001 | 0.001 | 0.000 | 0.001 | 0.001 | 0.000 | 0.002 | 0.000 | 0.000 | 0.000 | 0.007 | 0.007 | 0.000 | 0.013 |
| Pb | 0.004 | 0.004 | 0.003 | 0.002 | 0.003 | 0.004 | 0.005 | 0.002 | 0.000 | 0.005 | 0.001 | 0.000 | 0.000 | 0.000 |
| Y | 0.889 | 0.872 | 0.883 | 0.903 | 0.906 | 0.914 | 0.863 | 1.249 | 1.273 | 1.429 | 1.303 | 1.202 | 1.167 | 1.162 |
| La | 0.000 | 0.000 | 0.000 | 0.000 | 0.001 | 0.000 | 0.000 | 0.020 | 0.030 | 0.024 | 0.032 | 0.017 | 0.043 | 0.026 |
| Ce | 0.000 | 0.001 | 0.003 | 0.000 | 0.000 | 0.001 | 0.000 | 0.093 | 0.124 | 0.100 | 0.122 | 0.080 | 0.162 | 0.107 |
| Pr | 0.001 | 0.000 | 0.000 | 0.000 | 0.000 | 0.000 | 0.001 | 0.015 | 0.024 | 0.018 | 0.028 | 0.017 | 0.032 | 0.023 |
| Nd | 0.001 | 0.002 | 0.000 | 0.002 | 0.000 | 0.001 | 0.000 | 0.090 | 0.133 | 0.119 | 0.153 | 0.122 | 0.200 | 0.148 |
| Sm | 0.004 | 0.003 | 0.001 | 0.002 | 0.001 | 0.002 | 0.001 | 0.037 | 0.043 | 0.043 | 0.076 | 0.059 | 0.066 | 0.070 |
| Eu | 0.000 | 0.000 | 0.000 | 0.000 | 0.000 | 0.000 | 0.000 | 0.004 | 0.019 | 0.006 | 0.005 | 0.001 | 0.027 | 0.000 |
| Gd | 0.016 | 0.016 | 0.008 | 0.011 | 0.009 | 0.008 | 0.009 | 0.058 | 0.051 | 0.063 | 0.093 | 0.079 | 0.067 | 0.087 |
| Dy | 0.037 | 0.036 | 0.038 | 0.036 | 0.037 | 0.038 | 0.037 | 0.101 | 0.077 | 0.096 | 0.096 | 0.084 | 0.076 | 0.083 |
| Ho | 0.012 | 0.013 | 0.010 | 0.010 | 0.009 | 0.009 | 0.009 | 0.040 | 0.033 | 0.036 | 0.054 | 0.041 | 0.036 | 0.047 |
| Er | 0.028 | 0.021 | 0.030 | 0.030 | 0.030 | 0.032 | 0.031 | 0.038 | 0.037 | 0.040 | 0.033 | 0.040 | 0.029 | 0.033 |
| Tm | 0.000 | 0.000 | 0.000 | 0.000 | 0.000 | 0.000 | 0.000 | 0.000 | 0.000 | 0.000 | 0.000 | 0.000 | 0.000 | 0.000 |
| Yb | 0.032 | 0.029 | 0.034 | 0.040 | 0.027 | 0.031 | 0.037 | 0.040 | 0.042 | 0.045 | 0.033 | 0.045 | 0.031 | 0.036 |
| Lu | 0.000 | 0.000 | 0.000 | 0.000 | 0.000 | 0.000 | 0.000 | 0.000 | 0.000 | 0.000 | 0.000 | 0.000 | 0.000 | 0.000 |
| P | 0.979 | 0.986 | 0.991 | 0.971 | 0.982 | 0.967 | 0.994 | 0.000 | 0.000 | 0.000 | 0.000 | 0.000 | 0.000 | 0.000 |
|  |  |  |  |  |  |  |  |  |  |  |  |  |  |  |

Supplementary Table S1 | Continued HREE minerals

|  |  | | | |  |  | |  |  | | |  | |
| --- | --- | --- | --- | --- | --- | --- | --- | --- | --- | --- | --- | --- | --- |
|  | Y(Ce)-Ca-silicate | | | |  | Ce(Y)-Ca-silicate | |  | Y-Fe-silicate-1 | | | Y-Fe-silicate-2 | |
| F | 1.61 | 2.64 | 2.36 | 1.82 | 1.75 | 1.23 | 1.83 | 1.93 | 0.29 | 0.35 | 0.20 | b.d | b.d |
| MgO | b.d | b.d | b.d | b.d | b.d | 1.23 | 1.23 | 1.19 | 0.10 | 0.10 | 0.10 | 0.01 | 0.01 |
| K_2_O | 0.02 | b.d | 0.004 | 0.02 | b.d | 0.04 | 0.04 | 0.1 | 0.05 | 0.05 | 0.06 | 0.03 | 0.06 |
| Na_2_O | b.d | b.d | b.d | b.d | b.d | b.d | b.d | b.d | b.d | b.d | 0.022 | b.d | b.d |
| FeO | 0.16 | 0.14 | 0.29 | 0.12 | 0.11 | 1.70 | 1.51 | 1.33 | 10.26 | 10.96 | 8.37 | 5.53 | 5.8 |
| SiO_2_ | 19.64 | 24.64 | 22.90 | 22.92 | 21.70 | 31.41 | 29.02 | 32.02 | 23.16 | 23.27 | 21.45 | 24.38 | 18.25 |
| CaO | 11.48 | 11.81 | 13.84 | 12.82 | 12.92 | 4.07 | 4.17 | 7.12 | 0.42 | 0.43 | 0.51 | 0.45 | 0.40 |
| MnO | 0.69 | 0.69 | 0.58 | 0.52 | 0.54 | b.d | b.d | b.d | b.d | b.d | b.d | b.d | b.d |
| Al_2_O_3_ | b.d | b.d | b.d | b.d | b.d | 0.16 | b.d | 0.17 | b.d | b.d | b.d | b.d | b.d |
| ThO_2_ | 0.05 | b.d | b.d | 0.06 | 0.01 | b.d | 0.03 | b.d | b.d | 0.04 | b.d | b.d | 0.04 |
| UO_2_ | b.d | 0.02 | b.d | b.d | 0.06 | b.d | b.d | b.d | 0.07 | 0.361 | 0.04 | b.d | b.d |
| PbO | 0.71 | 0.60 | 0.21 | 0.16 | 0.12 | b.d | 0.04 | 0.03 | 0.04 | 0.02 | b.d | 0.17 | 0.12 |
| Y_2_O_3_ | 16.58 | 17.58 | 20.63 | 21.7 | 21.65 | 5.35 | 4.96 | 5.03 | 10.29 | 5.02 | 9.69 | 18.47 | 20.5 |
| La_2_O_3_ | 2.60 | 2.44 | 2.15 | 2.28 | 2.13 | 3.80 | 4.34 | 3.61 | 2.95 | 3.04 | 2.95 | 0.42 | 0.53 |
| Ce_2_O_3_ | 11.24 | 10.59 | 8.55 | 8.83 | 8.65 | 16.59 | 18.47 | 15.78 | 13.92 | 13.43 | 13.61 | 4.20 | 3.62 |
| Pr_2_O_3_ | 2.52 | 2.54 | 2.08 | 2.28 | 1.92 | 4.16 | 4.23 | 3.44 | 3.11 | 3.19 | 1.57 | 1.17 | 1.29 |
| Nd_2_O_3_ | 12.02 | 11.39 | 10.39 | 10.69 | 10.36 | 17.28 | 16.88 | 13.39 | 13.79 | 14.52 | 14.59 | 10.9 | 9.79 |
| Sm_2_O_3_ | 3.26 | 3.24 | 3.26 | 3.22 | 2.85 | 3.0 | 3.13 | 2.47 | 3.28 | 3.45 | 2.85 | 4.38 | 4.01 |
| Eu_2_O_3_ | b.d | b.d | b.d | b.d | b.d | b.d | b.d | b.d | b.d | b.d | b.d | b.d | b.d |
| Gd_2_O_3_ | 4.10 | 3.92 | 3.68 | 3.74 | 3.95 | 3.27 | 3.32 | 2.53 | 4.05 | 1.64 | 3.75 | 5.33 | 5.24 |
| Dy_2_O_3_ | 3.40 | 3.25 | 3.85 | 3.78 | 3.79 | 1.67 | 1.64 | 1.09 | 2.33 | 2.49 | 3.02 | 3.99 | 4.76 |
| Ho_2_O_3_ | 1.09 | 1.26 | 1.34 | 1.21 | 1.48 | 1.16 | 0.69 | 0.64 | 0.97 | 1.13 | 1.04 | 1.97 | 1.92 |
| Er_2_O_3_ | 1.12 | 0.83 | 1.61 | 1.49 | 1.45 | 0.19 | 0.48 | 0.07 | 0.42 | 0.50 | b.d | 0.55 | 0.70 |
| Tm_2_O_3_ | 0.65 | 0.56 | 0.29 | 0.44 | 0.45 | 0.48 | b.d | 0.21 | 0.48 | 0.36 | 0.18 | 0.49 | 0.63 |
| Yb_2_O_3_ | 0.91 | 1.06 | 0.99 | 1.27 | 1.35 | 0.11 | b.d | b.d | 0.18 | b.d | b.d | 0.16 | 0.74 |
| Lu_2_O_3_ | 0.40 | 0.33 | 0.44 | 0.42 | 0.39 | 0.13 | b.d | 0.38 | 0.15 | 0.07 | 0.23 | 0.13 | 0.27 |
| P_2_O_5_ | 0.55 | 0.62 | 0.85 | 0.91 | 1.14 | 0.16 | 0.14 | 0.18 | 0.05 | b.d | b.d | 0.02 | b.d |
| SO_3_ | 0.32 | 0.55 | b.d | 0.16 | 0.40 | 1.55 | 3.47 | 5.51 | 0.70 | 2.19 | 0.83 | 0.81 | 1.83 |
| Cl | 0.05 | 0.04 | 0.04 | 0.05 | 0.02 | 0.07 | 0.06 | 0.10 | 0.09 | 0.07 | 0.09 | 0.06 | 0.09 |
| F,Cl=O | -0.69 | -1.12 | -1.0 | -0.78 | -0.74 | -0.53 | -0.78 | -0.84 | -0.14 | -0.16 | -0.10 | -0.01 | -0.02 |
| Total | 94.46 | 99.6 | 99.31 | 100.1 | 98.43 | 98.26 | 98.89 | 97.47 | 91.02 | 86.49 | 85.03 | 83.59 | 80.59 |
|  | *Cations per 13 (O,OH,F)* | | |  |  | *Cations per 13 (O,OH,F)* | | | *Cations per 16 (O,OH,F)* | | |  |  |
| F | 0.732 | 1.072 | 0.971 | 0.744 | 0.729 | 0.474 | 0.707 | 0.690 | 0.163 | 0.199 | 0.121 | 0.000 | 0.000 |
| Mg | 0.000 | 0.000 | 0.000 | 0.000 | 0.000 | 0.225 | 0.226 | 0.202 | 0.027 | 0.027 | 0.029 | 0.003 | 0.003 |
| K | 0.004 | 0.000 | 0.001 | 0.003 | 0.000 | 0.006 | 0.006 | 0.014 | 0.011 | 0.012 | 0.015 | 0.007 | 0.015 |
| Na | 0.000 | 0.000 | 0.000 | 0.000 | 0.000 | 0.000 | 0.000 | 0.000 | 0.000 | 0.000 | 0.013 | 0.000 | 0.000 |
| Fe | 0.019 | 0.015 | 0.031 | 0.013 | 0.012 | 0.173 | 0.154 | 0.126 | 1.526 | 1.646 | 1.337 | 0.839 | 0.973 |
| Si | 2.826 | 3.169 | 2.984 | 2.967 | 2.862 | 3.833 | 3.552 | 3.626 | 4.134 | 4.195 | 4.111 | 4.437 | 3.673 |
| Ca | 1.770 | 1.627 | 1.932 | 1.778 | 1.826 | 0.532 | 0.547 | 0.864 | 0.080 | 0.083 | 0.105 | 0.088 | 0.086 |
| Mn | 0.084 | 0.075 | 0.064 | 0.057 | 0.060 | 0.000 | 0.000 | 0.000 | 0.000 | 0.000 | 0.000 | 0.000 | 0.000 |
| Al | 0.000 | 0.000 | 0.000 | 0.000 | 0.000 | 0.023 | 0.000 | 0.000 | 0.000 | 0.000 | 0.000 | 0.000 | 0.000 |
| Th | 0.002 | 0.000 | 0.000 | 0.002 | 0.000 | 0.000 | 0.001 | 0.000 | 0.000 | 0.002 | 0.000 | 0.000 | 0.002 |
| U | 0.000 | 0.006 | 0.000 | 0.000 | 0.018 | 0.000 | 0.000 | 0.000 | 0.028 | 0.145 | 0.017 | 0.000 | 0.000 |
| Pb | 0.028 | 0.021 | 0.007 | 0.006 | 0.004 | 0.000 | 0.001 | 0.001 | 0.002 | 0.001 | 0.000 | 0.008 | 0.007 |
| Y | 1.267 | 1.201 | 1.427 | 1.491 | 1.516 | 0.347 | 0.322 | 0.302 | 0.975 | 0.480 | 0.986 | 1.785 | 2.191 |
| La | 0.138 | 0.116 | 0.103 | 0.109 | 0.103 | 0.171 | 0.196 | 0.150 | 0.194 | 0.202 | 0.208 | 0.028 | 0.039 |
| Ce | 0.592 | 0.498 | 0.408 | 0.418 | 0.417 | 0.741 | 0.827 | 0.654 | 0.909 | 0.886 | 0.954 | 0.280 | 0.267 |
| Pr | 0.132 | 0.119 | 0.099 | 0.107 | 0.092 | 0.185 | 0.188 | 0.142 | 0.202 | 0.209 | 0.109 | 0.077 | 0.094 |
| Nd | 0.618 | 0.523 | 0.483 | 0.494 | 0.488 | 0.753 | 0.738 | 0.542 | 0.879 | 0.935 | 0.999 | 0.709 | 0.704 |
| Sm | 0.162 | 0.144 | 0.146 | 0.144 | 0.130 | 0.126 | 0.132 | 0.096 | 0.202 | 0.214 | 0.188 | 0.275 | 0.278 |
| Eu | 0.000 | 0.000 | 0.000 | 0.000 | 0.000 | 0.000 | 0.000 | 0.000 | 0.000 | 0.000 | 0.000 | 0.000 | 0.000 |
| Gd | 0.196 | 0.167 | 0.159 | 0.160 | 0.173 | 0.132 | 0.135 | 0.095 | 0.240 | 0.098 | 0.238 | 0.322 | 0.350 |
| Dy | 0.157 | 0.134 | 0.161 | 0.157 | 0.161 | 0.066 | 0.065 | 0.040 | 0.134 | 0.144 | 0.186 | 0.234 | 0.308 |
| Ho | 0.049 | 0.051 | 0.055 | 0.049 | 0.061 | 0.044 | 0.027 | 0.023 | 0.054 | 0.064 | 0.063 | 0.113 | 0.121 |
| Er | 0.051 | 0.034 | 0.066 | 0.061 | 0.060 | 0.007 | 0.018 | 0.002 | 0.024 | 0.028 | 0.000 | 0.031 | 0.044 |
| Tm | 0.029 | 0.022 | 0.012 | 0.018 | 0.018 | 0.018 | 0.000 | 0.007 | 0.027 | 0.020 | 0.011 | 0.028 | 0.039 |
| Yb | 0.040 | 0.042 | 0.039 | 0.050 | 0.054 | 0.004 | 0.000 | 0.000 | 0.010 | 0.000 | 0.000 | 0.009 | 0.045 |
| Lu | 0.017 | 0.013 | 0.017 | 0.016 | 0.016 | 0.005 | 0.000 | 0.013 | 0.008 | 0.004 | 0.013 | 0.007 | 0.016 |
| P | 0.167 | 0.168 | 0.234 | 0.249 | 0.318 | 0.041 | 0.036 | 0.043 | 0.019 | 0.000 | 0.000 | 0.008 | 0.000 |
| S | 0.035 | 0.053 | 0.000 | 0.016 | 0.040 | 0.142 | 0.319 | 0.468 | 0.094 | 0.296 | 0.119 | 0.111 | 0.276 |
| Cl | 0.012 | 0.009 | 0.009 | 0.011 | 0.004 | 0.014 | 0.012 | 0.019 | 0.027 | 0.021 | 0.029 | 0.018 | 0.031 |
|  |  |  |  |  |  |  |  |  |  |  |  |  |  |
|  |  |  |  |  |  |  |  |  |  |  |  |  |  |

**Note**: For calculating the empirical formulae, Y-silicate is potentially related to keiviite-(Y), Y-Ca-silicate related to britholite-(Y), Ce(Y)-Ca-silicate related to

kainosite-(Ce), and Y-Fe-silicate related to rowlandite-(Y). Low totals of Y-silicate and Y-Fe-silicate suggest possibly metamict and hydrated.

" n.a" not analyzed

"b.d" below detection limits

Supplementary Table S2 | SIMS U-Pb and Th-Pb data from monazites in carbonatites

|  |  |  |  |  |  |  |  |  |
| --- | --- | --- | --- | --- | --- | --- | --- | --- |
| Sample | Th(ppm) | Th/U | ^208^Pb/^232^Th | ^208^Pb/^232^Th age (Ma) | Age Err (Ma) | ^206^Pb/^238^U | ^206^Pb/^238^U age (Ma) | Age Err (Ma) |
| NQ-01 | 5077 | 19 | 0.0103 | 208 | 4 | 0.0338 | 214 | 5 |
| NQ-02 | 6161 | 35 | 0.0107 | 215 | 3 | 0.0334 | 212 | 6 |
| NQ-03 | 3818 | 20 | 0.0106 | 213 | 4 | 0.0327 | 208 | 5 |
| NQ-04 | 5382 | 61 | 0.0100 | 201 | 3 | 0.0373 | 236 | 7 |
| NQ-06 | 5727 | 56 | 0.0100 | 201 | 3 | 0.0348 | 221 | 11 |
| NQ-07 | 3170 | 28 | 0.0108 | 217 | 4 | 0.0329 | 209 | 5 |
| NQ-08 | 6769 | 41 | 0.0110 | 222 | 4 | 0.0350 | 222 | 13 |
| NQ-09 | 5881 | 46 | 0.0109 | 219 | 4 | 0.0343 | 218 | 8 |
| NQ-10 | 2943 | 40 | 0.0110 | 220 | 4 | 0.0346 | 219 | 12 |
| NQ-12 | 1372 | 10 | 0.0103 | 207 | 3 | 0.0317 | 201 | 5 |
| NQ-14 | 5681 | 32 | 0.0103 | 208 | 3 | 0.0334 | 212 | 7 |
| NQ-15 | 5939 | 40 | 0.0110 | 220 | 3 | 0.0333 | 211 | 5 |
| NQ-16 | 4537 | 30 | 0.0107 | 214 | 4 | 0.0320 | 203 | 4 |
| NQ-17 | 7768 | 41 | 0.0112 | 226 | 3 | 0.0331 | 210 | 6 |
| NQ-18 | 3589 | 31 | 0.0111 | 224 | 4 | 0.0343 | 218 | 6 |
| NQ-20 | 6462 | 36 | 0.0106 | 214 | 4 | 0.0302 | 192 | 5 |
| NQ-21 | 6504 | 43 | 0.0104 | 209 | 3 | 0.0336 | 213 | 5 |
|  |  |  |  |  |  |  |  |  |

Supplementary Table S3∣LA-ICPMS analysis (ppm) of fluid inclusions in carbonatitic calcites

|  |  |  |  |  |  |  |  |  |  |  |  |  |
| --- | --- | --- | --- | --- | --- | --- | --- | --- | --- | --- | --- | --- |
| Sample | Li | Na | K | Zn | As | Rb | Mo | Cs | Ba | Pb | Cl | Br |
|  |  |  |  |  |  |  |  |  |  |  |  |
| NQ-02 | 151 | 68117 | 28162 | 987 | b.d | 129 | b.d. | 25.0 | 1522 | 2278 | 143594 | b.d |
| NQ-02 | 220 | 61468 | 45893 | 2287 | 203 | 277 | b.d | 111 | 3569 | 6728 | 138273 | 1482 |
| NQ-02 | 198 | 69429 | 24664 | 12175 | b.d | 177 | b.d | 61.0 | 2054 | 30075 | 124827 | b.d |
| NQ-05 | 120 | 65222 | 35882 | 1109 | 105 | 270 | b.d. | 87.0 | 2311 | 3196 | 147437 | 947 |
| NQ-05 | 149 | 66669 | 32023 | 733 | b.d | 134 | b.d. | 22.0 | 3012 | 2727 | 120521 | 1268 |
| NQ-05 | 123 | 66803 | 31667 | 836 | b.d | 112 | 16.0 | 27.0 | 470 | 3021 | 146647 | b.d |
| NQ-05 | 120 | 66038 | 33706 | 868 | 76.0 | 208 | 17.0 | 60.0 | 902 | 2708 | 161792 | b.d |
| NQ-05 | 118 | 66904 | 31396 | 710 | b.d | 183 | b.d | 37.0 | 24192 | 2639 | 135963 | b.d |
|  |  |  |  |  |  |  |  |  |  |  |  |  |
|  |  |  |  |  |  |  |  |  |  |  |  |  |

**Note**: "b.d" below detection limit

Supplementary Table S4 | LA-ICPMS analyses (ppm) of fluorapatites in carbonatites

|  |  |  |  |  |  |  |  |  |  |  |  |  |  |  |  |
| --- | --- | --- | --- | --- | --- | --- | --- | --- | --- | --- | --- | --- | --- | --- | --- |
|  | 1 | 2 | 3 | 4 | 5 | 6 | 7 | 8 | 9 | 10 | 11 | 12 | 13 | 14 | 15 |
| Rb | 0.28 | 0.22 | 0.28 | 0.26 | 0.54 | 1.62 | 0.27 | 0.30 | 0.31 | 0.34 | 0.16 | 0.12 | 0.31 | 0.22 | 0.25 |
| Ba | 1.57 | 0.89 | 2.59 | 6.89 | 2.49 | 3.10 | 7.83 | 128 | 9.51 | 1.4 | 0.88 | 4.23 | 76.2 | 1.05 | 1.30 |
| Th | 4.67 | 4.44 | 10.7 | 3.95 | 7.57 | 18.0 | 6.98 | 8.98 | 8.67 | 4.55 | 4.0 | 1.70 | 17.8 | 5.01 | 12.0 |
| U | 4.75 | 5.91 | 9.68 | 6.62 | 10.2 | 9.77 | 6.71 | 13.7 | 7.58 | 5.97 | 5.22 | 3.28 | 17.3 | 5.44 | 8.85 |
| Nb | 0.34 | 0.08 | 43.7 | 128 | 3.65 | 46.2 | 1.28 | 50.6 | 0.71 | 0.49 | 0.11 | 1.95 | 0.42 | 0.13 | 4.29 |
| Ta | b.d | b.d | b.d | b.d | b.d | b.d | b.d | b.d | b.d | b.d | b.d | b.d | b.d | b.d | b.d |
| Pb | 18.0 | 17.1 | 13.8 | 26.3 | 12.6 | 20.5 | 17.0 | 45.4 | 16.1 | 13.1 | 14.9 | 15.4 | 18.4 | 16.9 | 21.0 |
| Sr | 5644 | 5265 | 5113 | 5448 | 5135 | 5700 | 5386 | 5524 | 5444 | 5353 | 5289 | 5393 | 5781 | 5423 | 5643 |
| Zr | 0.12 | 0.07 | 0.34 | 0.76 | 0.25 | 0.45 | 0.13 | 0.67 | 0.10 | 0.08 | 0.04 | 0.05 | 0.23 | 0.04 | 0.11 |
| Hf | b.d | 0.08 | b.d | b.d | b.d | 0.09 | 0.09 | b.d | b.d | 0.11 | b.d | b.d | b.d | b.d | b.d |
| Y | 2119 | 1988 | 1798 | 1881 | 2194 | 1831 | 1809 | 1822 | 1854 | 1778 | 1860 | 1025 | 2186 | 1926 | 1946 |
| La | 900 | 551 | 477 | 831 | 292 | 1275 | 640 | 763 | 353 | 408 | 455 | 230 | 1208 | 711 | 1178 |
| Ce | 3016 | 1886 | 1537 | 2603 | 980 | 4004 | 2070 | 2407 | 1169 | 1345 | 1568 | 767 | 3869 | 2396 | 3936 |
| Pr | 512 | 335 | 260 | 430 | 177 | 632 | 353 | 396 | 213 | 237 | 283 | 138 | 621 | 415 | 659 |
| Nd | 3040 | 2062 | 1541 | 2465 | 1128 | 3429 | 2094 | 2252 | 1368 | 1463 | 1754 | 854 | 3443 | 2504 | 3774 |
| Sm | 857 | 643 | 453 | 683 | 386 | 865 | 610 | 627 | 466 | 470 | 569 | 281 | 895 | 731 | 1022 |
| Eu | 215 | 160 | 119 | 168 | 109 | 209 | 152 | 155 | 124 | 122 | 145 | 74.1 | 222 | 180 | 245 |
| Gd | 754 | 619 | 453 | 619 | 450 | 710 | 577 | 568 | 500 | 480 | 558 | 289 | 747 | 663 | 816 |
| Tb | 95.2 | 83.0 | 63.6 | 79.2 | 68.4 | 87.2 | 75.4 | 74.8 | 70.7 | 66.8 | 75.7 | 39.9 | 95.5 | 84.5 | 98.9 |
| Dy | 462 | 417 | 340 | 399 | 388 | 419 | 383 | 381 | 370 | 351 | 386 | 206 | 468 | 415 | 463 |
| Ho | 79.1 | 73.0 | 64.3 | 69.7 | 77.5 | 70.9 | 67.9 | 67.5 | 67.8 | 65.3 | 68.6 | 37.2 | 80.1 | 71.1 | 76.8 |
| Er | 179 | 168 | 157 | 160 | 196 | 159 | 158 | 160 | 159 | 157 | 159 | 87.7 | 186 | 164 | 170 |
| Tm | 21.4 | 20.0 | 19.6 | 19.6 | 25.3 | 18.6 | 18.6 | 19.7 | 19.5 | 19.3 | 19.0 | 10.7 | 22.5 | 19.2 | 19.7 |
| Yb | 125 | 113 | 118 | 113 | 157 | 103 | 108 | 113 | 116 | 112 | 109 | 63.4 | 131 | 108 | 108 |
| Lu | 13.9 | 12.6 | 14 | 12.7 | 18.2 | 11.4 | 12.1 | 12.8 | 13.4 | 12.8 | 12.3 | 7.21 | 15.0 | 12.1 | 11.8 |
|  |  |  |  |  |  |  |  |  |  |  |  |  |  |  |  |

**Note**: "b.d" below detection limit

Supplementary Table S5 | Sulfur isotopes of mineral phases in carbonatites

|  |  |  | | | |
| --- | --- | --- | --- | --- | --- |
| Sample | Mineral assemblage | ^34^S_CDT_ of S-bearing mineral separates (‰) | | | |
|  |  | Mo | Py | Ga | Brt |
| NQ-02 | Cc+Py+Ga+Mo | -6.7 | -7.0 | -8.8 |  |
| NQ-03 | Cc+Py+Kf |  | -6.6 |  |  |
| NQ-05a | Cc+Brt+Ga+Mo | -7.5 |  | -8.9 | +4.7 |
| NQ-05b | Cc+Brt+Ga+Mo | -7.7 |  | -10.7 | +5.1 |
| NQ-06 | Cc+Q+Ga |  | -7.1 | -11.1 |  |
| NQ-09 | Cc+Brt+Ga | -7.2 |  | -9.2 | +4.7 |
| NQ-11a | Cc+Py+Mo | -7.3 | -7.2 |  |  |
| NQ-12 | Cc+Brt+Py |  | -7.1 |  | +4.8 |
| NQ-15 | Cc+Q+Kf+Py |  | -6.8 |  |  |
| NQ-17 | Cc+Brt+Mo+Ga |  |  | -9 | +4.7 |
| NQ-18 | Cc+Brt+Ga+Q+Mo |  |  | -10.5 | +4.6 |
|  |  |  |  |  |  |
|  |  |  |  |  |  |

**Note**: Cc, calcite; Q, quartz; Py, pyrite; Ga, galena

Mo, molybdenite; Kf, K-feldspar; Brt, barite
